# A Molecular Dynamics Study of Mechanical and Conformational Properties of Conjugated Polymer Thin Films

**DOI:** 10.1021/acs.macromol.4c00232

**Published:** 2024-05-20

**Authors:** Yang Wang, Zhaofan Li, Kangmin Niu, Wenjie Xia, Andrea Giuntoli

**Affiliations:** † Zernike Institute for Advanced Materials, University of Groningen, 9747 AG, Groningen, The Netherlands; ‡ School of Materials Science and Engineering, University of Science and Technology Beijing, Beijing 100083, China; ¶ Department of Aerospace Engineering, Iowa State University, Ames, Iowa 50011, United States

## Abstract

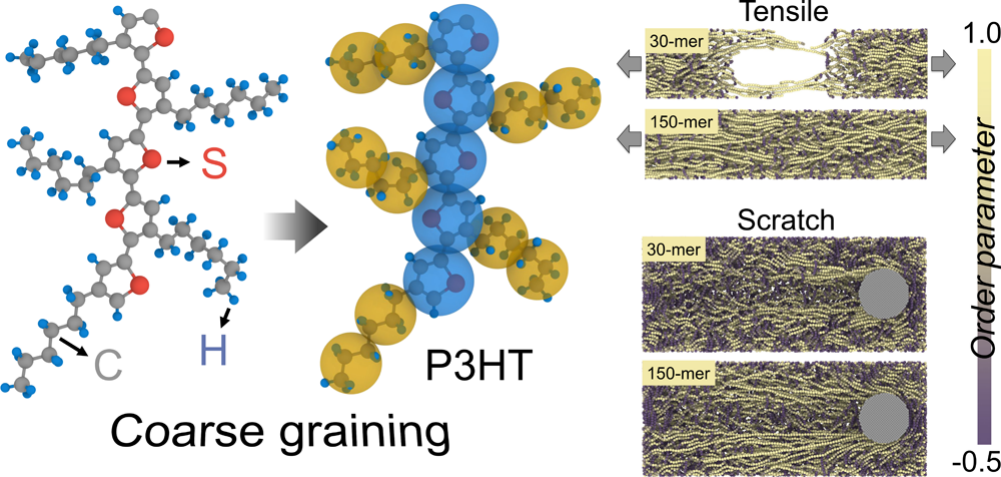

Understanding and predicting the mechanical and conformational properties of conjugated polymer (CP) thin films are a central focus in flexible electronic device research. Employing molecular dynamics simulations with an architecture-transferable chemistry-specific coarse-grained (CG) model of poly(3-alkylthiophene)s (P3ATs), developed by using an energy renormalization approach, we investigate the mechanical and conformational behavior of P3AT thin films during deformation. The density profiles and measures of local mobility identify a softer interfacial layer for all films, the thickness of which does not depend on *M*_*w*_ or side-chain length. Remarkably, Young’s modulus measured via nanoindentation is more sensitive to *M*_*w*_ than for tensile tests, which we attribute to distinct deformation mechanisms. High-*M*_*w*_ thin films show increased toughness, whereas longer side-chain lengths of P3AT resulted in lower Young’s modulus. Fractures in low-*M*_*w*_ thin films occur through chain pullout due to insufficient chain entanglement and crazing in the plastic region. Importantly, stretching promoted both chain alignment and longer conjugation lengths of P3AT, potentially enhancing its electronic properties. For instance, at room temperature, stretching P3HT thin films to 150% increases the conjugated length of P3HT thin films from 2.7 nm to 4.7 nm, aligning with previous experimental findings and all-atom simulation results. Furthermore, high-*M*_*w*_ thin films display elevated friction forces due to the chain accumulation on the indenter, with negligible variations in the friction coefficient across all thin film systems. These findings offer valuable insights that enhance our understanding and guide the rational design of CP thin films in flexible electronics.

## Introduction

Conjugated polymers (CPs) are functional macromolecules that generally contain a rigid conducting backbone, enabling charge transformation, and a flexible side chain promoting solubility during solution processing. The corresponding thin films have gained significant attention in the field of optoelectronic devices,^1^ light-emitting diodes (LEDs),^2,3^ and organic field-effect transistors (OFETs)^4^ due to their remarkable electronic, optical, and mechanical compliance properties. Among all CPs, poly(3-alkylthiophene)s (P3ATs) have gained significant attention due to their exceptional properties, including high charge carrier mobility, excellent processability, thermal stability, and availability in bulk quantities.^5−8^ Although the electronic performance of these films has traditionally been the primary focus, the thermomechanical properties of CP thin films, such as elasticity, flexibility, toughness, and stability, also play a crucial role in determining their overall device performance and reliability.^9,10^ Therefore, understanding and optimizing the mechanical behavior of these films are of utmost importance for the successful integration of conjugated polymers into electrically active applications, especially when the system is scaled down to the nanometer.

The mechanical properties, including modulus, stiffness, and toughness, are important for conjugated polymers in flexible device applications, although they are not easy to measure using conventional techniques due to the ultrathin structures and soft textures. Gu et al.^11−13^ and Lipomi et al.^14,15^ studied the mechanical properties of free-standing CP thin films based on the *film-on-water* and *film-on-elastomer* methods, where the thin film is deformed floating on the water and coating on an elastic substrate, respectively. Through backbone- and side-chain-engineering strategies, they found that longer alkyl side-chain length will soften the mechanical response of the CP thin film, while a more bulky backbone moiety will stiffen the polymer and further enhance the elastic modulus of the film. Xiang et al.^16^ explored the intrinsic mechanical properties of polymeric semiconductors using nanoindentation tests, revealing that the strong backbone rigidity can improve their strength and elastic behavior, while the long side chains may effectively increase the side-chain entanglements and interpenetration and further strengthen viscoelastic behavior. Meanwhile, a short side-chain length system leads to larger unrecoverable deformations and lower modulus and hardness due to easier interchain viscous slippage. Bao et al.^9,17^ emphasized and summarized the mechanical properties of stretchable polymer semiconductors, revealing that a rational molecular design strategy (i.e., *M*_*w*_, regioregularity, structural modifications in the polymer backbone, and side chain) could improve both electrical and mechanical properties simultaneously. However, due to the endless possibilities of CPs’ chemistry combining with their architectures, experimental measurement of mechanical properties for new designs of CPs can be time-consuming. Hence, a computational modeling and simulation framework to understand and predict the mechanical properties of CP thin film from a theoretical perspective can speed up the design of next-generation electronic devices.

Coarse-grained (CG) MD simulations have emerged as an effective approach to overcome the spatiotemporal limitations of traditional all-atomistic (AA) simulations by treating a group of atoms as one super CG bead. However, traditional CG models always overestimate the dynamics and underestimate the mechanical response of the system due to the smooth energy landscape and the loss of configurational entropy (*s*_*c*_) upon coarse-graining.^18,19^ Huang et al.^20^ and Lee et al.^21^ previously developed the P3HT CG model using a three-site and one-site mapping scheme, providing insightful studies on the phase separation and morphologies of a P3HT/C60 mixture. However, the one-site P3HT model exhibited overestimated density and softer mechanical response compared with the experiment, and the three-site model showed deviated diffusion behavior under different temperatures compared with the target AA model, necessitating the accurate CG modeling of conjugated polymer.^22,23^ To address these issues, we utilized the energy-renormalization (ER) approach in our recent work to systematically develop a temperature- and architecture-transferable CG model of P3ATs.^24−26^ Specifically, by varying the cohesive interaction parameter ϵ and effective distance parameter σ of Lennard-Jones (LJ) potential in a temperature-dependent style, the ER method could compensate the *s*_*c*_ loss of the system via renormalizing the system’s enthalpy (i.e., “entropy–enthalpy compensation” effect) and reproduce the AA density and dynamics over a wide temperature range.

In the present work, following our previous ER coarse-graining framework,^24,25^ we systematically explore the mechanical and conformational properties of P3AT thin film upon deformation using the chemistry-specific CG molecular dynamics simulation. Density and local mobility measurements showed a soft interfacial layer for all P3AT thin films with a thickness that does not depend on *M*_*w*_ or side-chain length. For each thin film system, the interior region shows stronger local stiffness and slower dynamics than that of the surface region. It is noted that Young’s modulus measured via nanoindentation is more sensitive to *M*_*w*_ than for tensile tests due to the distinct deformation mechanisms. High-*M*_*w*_ thin films showed increased toughness, whereas longer side-chain lengths result in a lower Young’s modulus, which is inversely correlated with the Debye–Waller factor. Fractures in low-*M*_*w*_ thin films occurred through chain pullout due to insufficient chain sliding and were accompanied by decreased chain orientation. Additionally, stretching prompted conspicuous chain alignment along the stretching direction and an increased conjugation length, which shows the potential to improve the electrical properties of the thin film and align with previous experimental and all atomistic simulation results. Finally, we conducted a scratch test of the P3AT thin film, revealing high friction and normal force in the indenter of the thin film with high *M*_*w*_ due to the adhesion of long chains to the indenter.

## Modeling and Simulation Procedure

### Simulation Model Setup

**Figure 1:**
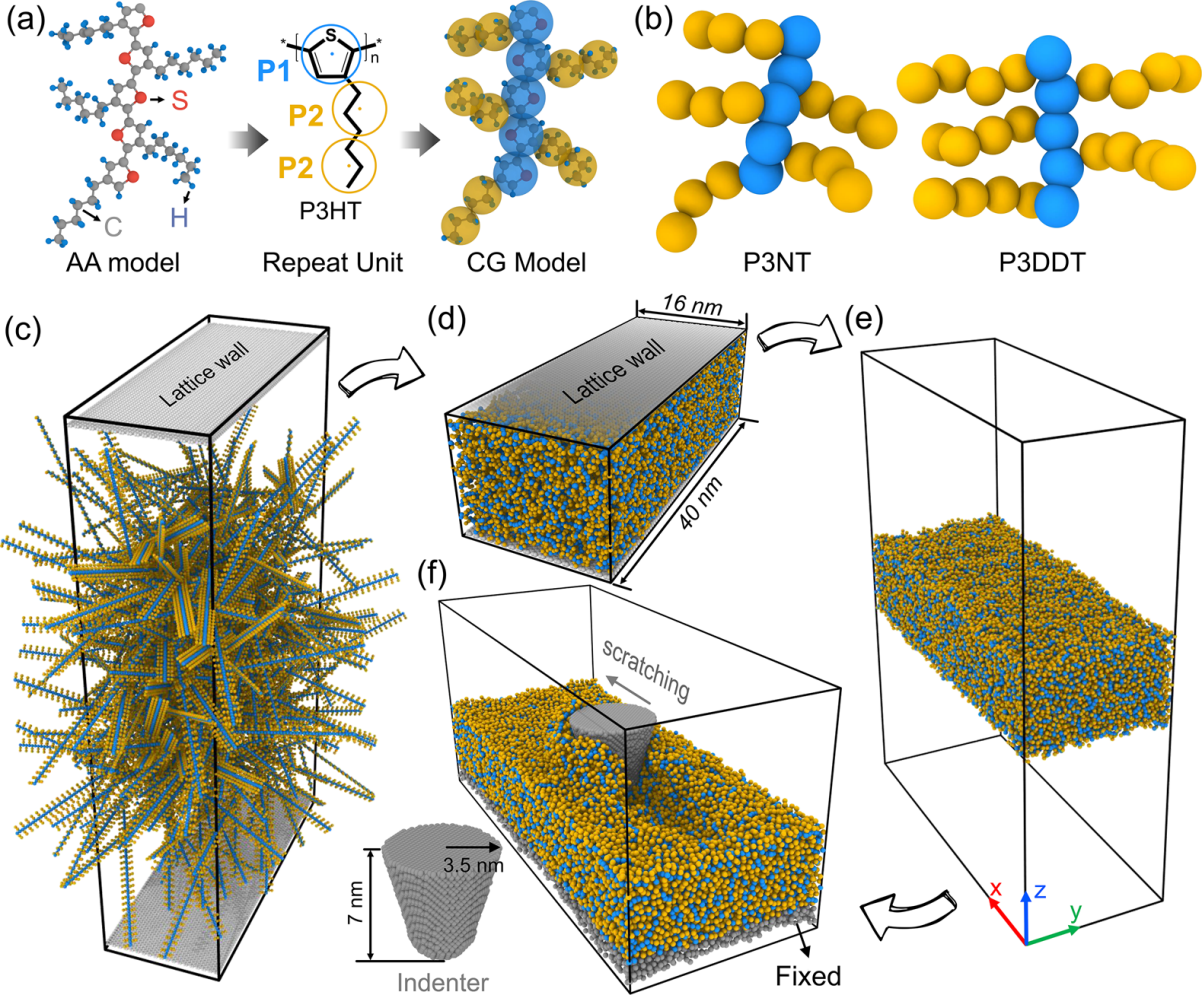
(a) Coarse-graining (left) P3HT all-atomistic (AA) model to the (right) coarse-grained (CG) model with the middle panel showing the CG mapping scheme. (b) The CG models of the P3AT derivative with different side-chain lengths, i.e., three (P3NT) and four (P3DDT) P2 beads per side chain. (c–f) Fabrication of the P3AT conjugated polymer thin film. The lattice walls are rigid and used to confine polymer chains from state (c) to (d). In panel (f), the 2 nm of the bottom polymer layer is treated as a rigid region (gray atoms), and the cone-shaped indenter is colored gray.

**Figure 2:**
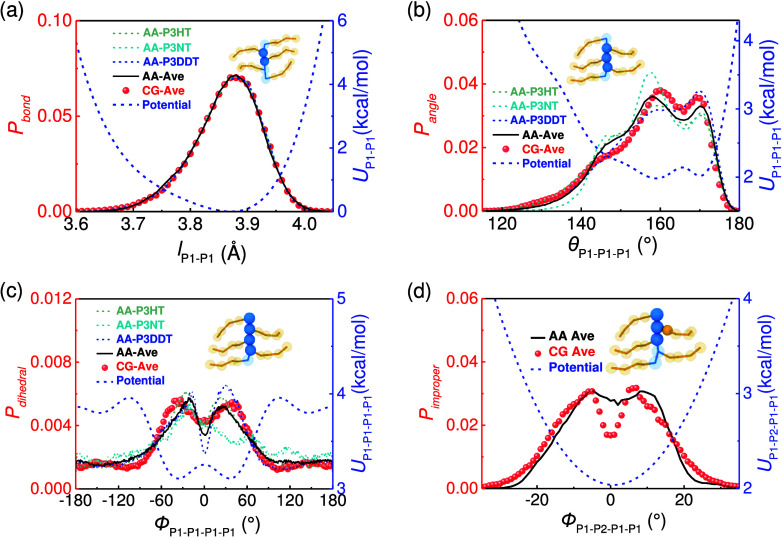
Representative bonded probability distributions of the (a) P1–P1 bond, (b) P1–P1–P1 angle, (c) P1–P1–P1-P1 dihedral, and (d) P1–P2–P1–P1 improper terms of P3HT, P3NT, and P3DDT AA and CG (symbol) models. The solid black line denotes the averaged distribution from AA simulations of P3HT, P3NT, and P3DDT. The blue dashed lines denote the relative CG potentials, and the insets show the corresponding bonded interaction form.

The atomistically informed P3AT CG model utilized in this work is depicted in Figure 1a, where each monomer is represented by two CG bead types, i.e., P1 and P2. Note that the bonded interactions are reparameterized by incorporating P3ATs with different side-chain lengths based on our previous work.^25^ Namely, we consider three derivatives of P3AT, where A = hexyl, nonyl, and dodecyl, i.e., P3HT (2 beads per side chain), P3NT (3 beads per side chain), and P3DDT (4 beads per side chain), as shown in Figure 1a,b. The force center is located at the center of mass of the underlying atoms of the CG bead. Built upon the defined CG mapping scheme, we derived the bonded interactions of P3ATs with different side-chain lengths by using iterative Boltzmann inversion (IBI).
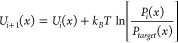
1where *k*_*B*_ is the Boltzmann constant and *T* is the absolute temperature; the variable *x* refers to the bond, angle, dihedral, and improper terms, respectively. *P*_*i*_(*x*) is the probability distribution of the relative bonded term in the *i*th iteration; *P*_*target*_(*x*) is the target average probability distribution of the relative bonded term from the AA simulations of P3HT, P3NT, and P3DDT (black solid line in Figure 2). The detailed AA and CG simulations for obtaining the bonded and nonbonded interactions can be found in the Supporting Information and previous work.^25^

The representative bonded probability distributions are shown in Figure 2. After 4–7 iterations of IBI, the bonded distribution of the CG model (symbol) is consistent with the target AA model, and the corresponding CG potentials are shown in the right *y*-axis in Figure 2. Borrowing the idea of ER, we characterized the nonbonded interactions (Gromacs style LJ potential) of the CG P3AT model by capturing the density and dynamics of the CG model to be consistent with the AA model over a wide temperature range and different architectures, addressing the issue that CG models are hard to transfer for other thermodynamic states and chemical structures. Specifically, we introduce temperature-dependent ER factors α(*T*) and β(*T*) to the nonbonded parameters of ϵ and σ in the LJ potential (eq 2). The initial nonbonded interaction parametrizations are obtained using the radial distribution function (RDF) between different bead species. The *S*_*LJ*_ denotes an additional switching function that ramps the energy and forces smoothly to zero between an inner cutoff *R*_*i*_ (12 Å) and outer cutoff *R*_*o*_ (15 Å), and the coefficients *A*, *B*, and *C* are computed by LAMMPS to perform the shifting and smoothing.^27^ All bonded and nonbonded parameters of the P3ATs CG model are given in Tables S1 and S2. For a more comprehensive understanding of the IBI and ER framework for coarse-graining P3AT, we encourage readers to refer to our recently published work.^24,25^ In the present work, we analyze the mechanical response of P3AT thin film at 300 K, so the LJ parameters of ϵ and σ for P1 and P2 beads are constant and can be determined using the equations in Table S2.
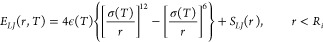
2
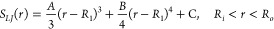
3

Next, we use two lattice rigid plates to represent the confinement walls while preparing the polymer thin film. The dimension of the two plane walls is 16 × 40 nm (Figure 1c,d), and the initial distance between them is 200 nm to avoid excessive polymer overlapping. Then, the P3AT chains with different *M*_*w*_ (chain lengths, *n*) and side-chain lengths are randomly packed into the space between rigid walls by using an in-house Python script (Figure 1c). Herein, we aim to explore the effect of *M*_*w*_ (i.e., chain length) on mechanical property, so the total number of polymer CG beads in each system is kept consistent (around 60 000) by tuning the chain numbers to guarantee the same thin film thickness. For P3AT with different side-chain lengths, we choose a *M*_*w*_ similar to the 80-mer P3HT thin film system, and the chain lengths for P3NT and P3DDT are determined as 62 and 52 monomers, respectively. Detailed information on all P3AT thin film systems in the current work is summarized in Table 1. An FCC crystal diamond with a conical shape is used to indent and scratch the polymer thin film. The detailed information about the nanoindenter is depicted in Figure 1f. The nonbonded interaction parameters of the indenter are 0.066 kcal/mol and 3.4 Å.^28^ And the nonbonded interactions between the polymer and indenter are described using the Lorenz–Berthelot mixing rule (*i* and *j* denote the polymer and indenter, respectively), which is commonly used in the polymer nanocomposites:^29,30^
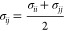
4
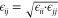
5

**Table 1:** Detailed Information on P3AT Thin Film Systems with Different *M*_*w*_ (or Chain Length, *n*) and Side-Chain Lengths^a^

polymer system	chain length, *n* (monomer)	*M*_*w*_ (kDa)	chain number	total number of beads	effective thickness (nm)
P3HT	10	2.963	2000	60 000	8.24
	30	8.890	666	59 940	8.14
	50	14.816	400	60 000	8.21
	80	23.705	250	60 000	8.12
	100	29.632	200	60 000	8.13
	150	44.448	133	59 850	8.17
P3NT	62	23.463	241	59 768	8.11
P3DDT	52	23.949	230	59 800	8.25

a The chain number is tuned to keep a nearly constant total number of beads in each system.

### Simulation Details

To equilibrate the free-standing P3AT thin film system, a randomly distributed initial velocity based on a temperature of 800 K is used for the polymer to initialize the structure. The time step is 4 fs, and the periodic boundary condition (PBC) is used for all three directions. Then, the systems are energy minimized using the iterative conjugant gradient algorithm.^31^ To eliminate the bead overlapping and get a fully relaxed system, we use a pure repulsive potential to describe the interaction between P3AT chains and between the walls and P3AT chains. During the simulation, the walls are treated as a rigid body and can only move along the *z*-axis. The polymer chains are relaxed under the NPT ensemble with a temperature of 800 K and pressure of 1 atm for the *x* and *y* directions and 200 atm for the *z* direction for 2 ns, yielding the system shown in Figure 1d. After that, we remove the high pressure in the *z*-direction and relax the system with the same simulation conditions as in the previous stage for 2 ns. Next, the *real* LJ potential replaces the pure repulsive potential between polymers to condense the film, and pure repulsive energy is held for describing the interaction between the lattice walls and polymer. Afterward, we remove the lattice walls, extend 20 nm above and below the film, and then use non-PBC in the *z*-direction (Figure 1e), ensuring that the periodic images of the resulting slab do not interact with one another. The system is then annealed at NVT ensemble by ramping the temperature from 300 to 800 K two times at a rate of 0.5 K/ps and with a total time of 4.5 ns. The temperature-damping parameter of *T*_*damp*_ is 400 fs, which is specified in time units and relaxes the temperature in a period of roughly 400 fs, representing how rapidly the temperature is relaxed. The tensile mechanical property of the free-standing thin film (Figure 1e) is tested first. Then we impart the indenter with an initial velocity of 0.05 nm/ps to progressively embed it into the polymer thin film until it reaches a final depth of 30 Å (40 Å far away from the fixed substrate), which is greatly larger than the cutoff distance and considered to have no interaction with the substrate. Subsequently, we withdraw the indenter from the polymer matrix at the same velocity to obtain the indentation curves. As for the scratching test, the indenter is initially embedded in the film with a depth of 30 Å (Figure 1f), and then the indenter scratches over 300 Å with a velocity of 0.05 nm/ps. Three independent runs are performed for each deformation condition to improve the statistics.

### Properties’ Calculation

To characterize the chain dynamics of the thin film under the effect of the free surface, we calculate the mean square displacement (MSD), , of the CG beads in a specific layer of the thin film along the *z*-axis:
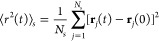
6where *r*_*j*_(*t*) is the position of the *j*th bead at time *t*, and the ⟨···⟩ denotes the ensemble average of *N*_*s*_ beads in the *s*th layer of the thin film. The dynamical heterogeneity of the P3ATs thin films is characterized using the short-time fast dynamics property of the Debye–Waller factor (DWF, ⟨*u*^2^⟩), which is determined as the MSD value at the picosecond time scale and chosen as *t* = 4 ps here, i.e., ⟨*u*^2^⟩ = ⟨*r*^2^(4)⟩.^18,24^

A strain-controlled uniaxial tension deformation is performed in the *x*-direction for the thin film systems to calculate the elastic modulus. The atomic virial stress tensor calculates the stress component in the tensile direction.
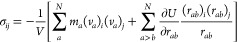
7where *V* is the system volume at the equilibrium stage and normalized with the thickness of the polymer thin film, *N* is the total number of particles in the system, *r*_*ab*_ stands for the distance between particles *a* and *b*, *U* represents the total energy of the system, and *m*_*a*_ and *v*_*a*_ are the mass and velocity of the particle, respectively. Specifically, the equation for the *i*, *j* components (where *i* and *j* = *x*, *y*, *z*) stands for the six-element symmetric stress tensor, and the tensile stress and *E* in this work are determined using the σ_*xx*_ component (tensile direction) due to the anisotropic geometry of the thin film. The constant strain rate is 0.5 ns^–1^, which is greatly larger than the experimental value due to the current computational limitation of the MD technique but lies in the range of values that are widely adopted in previous works.^24,32−34^ Young’s modulus is derived from the linear fit of the elastic stage of the stress–strain curve within 4% strain. To quantify the local atomic stress distribution, we introduce the Von Mises stress for each CG bead by considering their Voronoi volume, using eq 8:
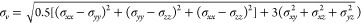
8

During the tensile and scratch deformation, we evaluate the polymer chain orientation of the P3AT using the orientation order parameter *P*_2_:
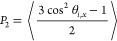
9where θ_*i*,*x*_ is the angle between an arbitrary bond vector *i* and the deformation direction *x* and ⟨···⟩ denotes the ensemble average. The simulation is performed in the open-source large-scale atomic-molecular massively parallel simulator (LAMMPS),^35^ and the visualization is achieved with OVITO.^36^ The MDAnalysis (www.mdanalysis.org),^37^ an object-oriented Python toolkit, is used to analyze molecular dynamics trajectories.

## Results and Discussion

### Thin Film Characterization

**Figure 3:**
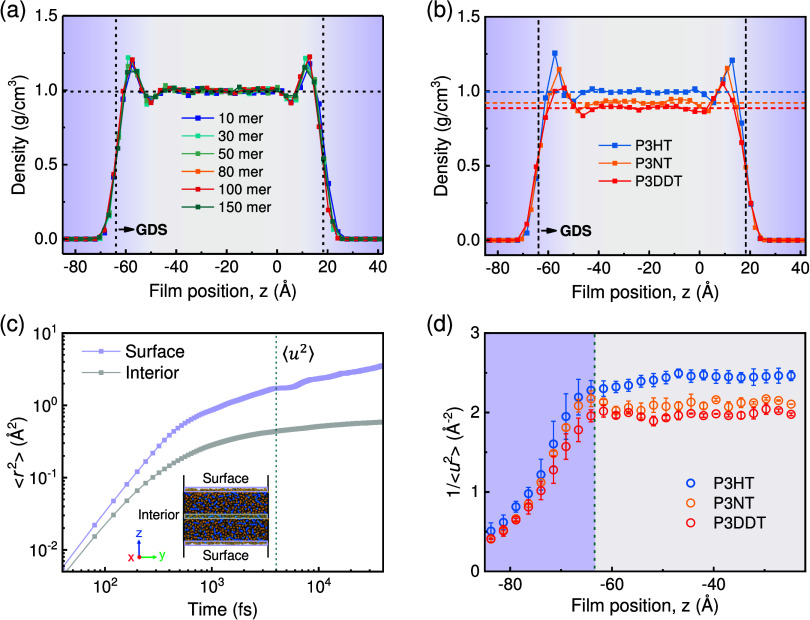
Density profile along the *z*-axis for the (a) P3HT thin film with different *M*_*w*_ ranging from 2.963 to 44.448 kDa and (b) P3AT thin films with the same *M*_*w*_ but different side-chain lengths. In panels (a) and (b), the vertical dashed lines denote the Gibbs dividing surface for determining the film’s effective thickness, and the horizontal dashed lines reveal the average density of polymer in the interior region. (c) MSD ⟨*r*^2^⟩ vs time for the interior and surface layer of the P3HT thin film system with a chain length of *n* = 80. The Debye–Waller factor ⟨*u*^2^⟩ is determined as the MSD value when time equals 4 ps, i.e., ⟨*u*^2^⟩ = ⟨*r*^2^(4)⟩. (d) Spatial distribution of the local stiffness, 1/⟨*u*^2^⟩, as a function of polymer *z* position for P3HT (80 mer), P3NT (62 mer), and P3DDT (52 mer) thin film systems with the same *M*_*w*_; only half of the thin film is shown here given the symmetry. The interfacial region is highlighted in blue and possesses low molecular stiffness compared to the interior region due to the effect of the free surface.

After film equilibration in Figure 1e, we divided the thin film into 50 sublayers along the *z*-direction (the inset of Figure 3c) to systematically characterize the local density and dynamics under the effect of the free surface. Figure 3a shows the density profile of the P3HT thin film with different *M*_*w*_ along the *z*-direction, where the effective thickness of the film can be determined using the Gibbs dividing surface (GDS) method:^38,39^
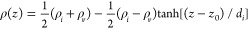
10where ρ_*i*_ and ρ_*v*_ represent the interior and vacuum densities, respectively, *d*_*i*_ is the thickness of the interface, and *z*_0_ is the positions of Gibbs’s dividing surface (vertical dashed lines in Figure 3a,b), which is also illustrated in Figure S1a. We characterize the density profiles of P3HT, P3NT, and P3DDT thin films with similar *M*_*w*_ values (Table 1) and different side-chain lengths. Figure 3a,b show a consistent interfacial position (*z*_0_ = −64.6 Å on one side) for all P3AT free-standing thin film systems regardless of *M*_*w*_ and side-chain length, and all P3AT thin films have an effective thickness of 8.1–8.2 nm, as summarized in Table 1. The plateau density observed in the interior region of different P3HT molecules with varying *M*_*w*_ is 0.996 ± 0.004 g/cm^3^, similar to both the experimentally and simulation-determined values of 0.936 and 0.955 g/cm^3^, respectively.^20,40^ The densities for interior P3NT and P3DDT thin films are 0.931 ± 0.010 and 0.889 ± 0.012 g/cm^3^ (horizontal dashed lines in Figure 3a,b), respectively, showing a decrease in density as the side-chain length of P3AT increases and consistent with previous MD works.^22,25^ It is worth noting that the heightened density peak observed in the profile of the free-standing thin film is attributed to the bottlebrush-like architecture exhibited by the P3AT. Figure S1d shows the density profiles of backbone and side-chain beads, respectively, revealing the pronounced peak for backbone beads. This phenomenon bears similarity to previous MD investigations on star polymer thin films, wherein the peak of the core density near the free surface is amplified with shorter arm lengths.^41,42^

Since Keddie et al.^43^ first observed a decrease in *T*_*g*_ in polymer thin films, extensive studies explored the effect of confinement on these systems, where substantial deviations are observed in relaxation dynamics,^44,45^ surface modulus,^46^ viscosity,^47^ and elastic modulus^48^ compared to their bulk counterparts. Near the film surfaces and interfaces, polymer chains experience altered mobility compared with those within the bulk. Herein, we calculated the MSD curve layer-by-layer for each system along the *z*-direction to explore the effects of the free surface, *M*_*w*_, and side-chain lengths on the dynamics of free-standing polymer thin film. Taking the P3HT thin film with an *M*_*w*_ of 23.705 kDa (*n* = 80) as an example system, we separately calculated the MSD curves of the interior and surface layer (Figure 3c). Results show a significant difference in dynamics between the interior and surface layer, where the interior polymer manifests a discernibly reduced mobility and slower dynamics compared to the surface counterpart. Similar to previous works,^49,50^ particles in the interior of the polymer film or near the substrate with strong interaction experience more constraints and interactions with neighboring atoms than on the surface, which can limit their mobility. Considering the distinct density profiles of backbone and side-chain beads (Figure S1d), we separately investigated their dynamics along the *z*-axis as demonstrated in Figures S2 and S3 in the Supporting Information. The results revealed that both backbone and side-chain beads exhibit significantly stronger dynamics near the surface compared to the interior region. Previous works have also suggested that the DWF ⟨*u*^2^⟩ could predict molecular relaxations and glassy dynamical heterogeneities in bulk and confined systems.^44,48,51,52^ ⟨*u*^2^⟩ is a fast dynamics physical property relating to the segmental “free volume” on the order of the picosecond time scale. In this work, ⟨*u*^2^⟩ is determined as the magnitude of the MSD at *t* = 4 ps, which corresponds to the onset of caging dynamics as we determined in our previous work.^18^ Accordingly, the local molecular stiffness (1/⟨*u*^2^⟩) of the polymer is inversely related to the DWF for harmonic vibration of segments within a cage surrounded by their neighbors, i.e., ⟨*u*^2^⟩ ∼ *k*_*B*_*T*/*k*, where *k*_*B*_ is Boltzmann’s constant and *k* is the local spring constant.^48^Figure 3d shows the local stiffness (1/⟨*u*^2^⟩) distribution along the *z*-axis of P3HT (80-mer), P3NT (62-mer), and P3DDT (52-mer) thin films. Similar to the density profile, we observe a clear difference in local stiffness in the interior and interfacial layers for all P3AT thin film systems, demonstrating the same interfacial position as that obtained from GDS results (*z* = −64.6 Å). Notably, increasing the side-chain length of P3AT causes a lower 1/⟨*u*^2^⟩ in each layer of the thin film, in agreement with previous work showing that the longer side-chain length accelerates the dynamics and induces a lower Young’s modulus in P3AT thin films.^53,54^ Additionally, the dynamics of the backbone and side-chain beads along the thickness direction are separately discussed in the Supporting Information.

### Uniaxial Tension and Nanoindentation

**Figure 4:**
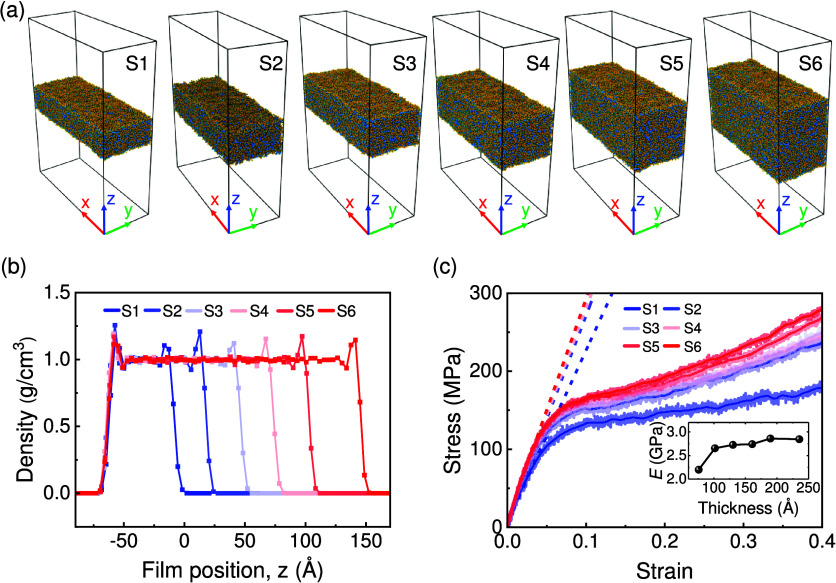
(a) Snapshots of equilibrated P3HT (80 mer) thin film systems with different thicknesses. (b) Density profile of the P3HT thin film (80 mer) with different thicknesses. (c) Stress–strain curves of the P3HT thin film at varying thickness with linear dashed lines indicating the linear fitting of the elastic stage of stress–strain curves. The inset shows the relationship between thickness and Young’s modulus of P3HT thin film.

To investigate the effect of the thickness on the mechanical properties of the polymer thin film, we use P3HT with 80 monomers per chain to create thin film systems with different thicknesses by adjusting the chain number. Detailed information for the P3HT system with different thicknesses is illustrated in Table 2. Figure 4a,b show the equilibrated P3HT thin film systems with different film thicknesses and relative density profiles along the *z*-axis. To calculate the film thickness, we measure the difference between the maximum and minimum *z*-coordinate values of all CG beads within the system, averaged over all configurations in the equilibrated state, which is different from the effective thickness obtained from the GDS method and is considered to calculate the σ_*xx*_. Figure 4c shows the stress–strain curves of P3HT thin films with different thicknesses with the inset exhibiting the relation between Young’s modulus and film thickness, revealing that as the thickness increases, the modulus progressively rises until it reaches a plateau, which is attributed to the reduced proportion of the interface layer in the thin film and consistent with the previous thin film work.^48^ Notably, the S2 system has a thickness of 10.17 nm and an effective thickness of 8.12 nm from the GDS method (Table 1).

**Table 2:** System Information and Young’s Modulus of P3HT Thin Films (80 Monomers per Chain) with Different Thicknesses

system label	chain number	thickness (nm)	Young’s modulus (GPa)
S1	166	7.60	2.200 ± 0.026
S2	250	10.17	2.654 ± 0.012
S3	333	13.02	2.726 ± 0.008
S4	416	16.08	2.741 ± 0.010
S5	500	18.95	2.860 ± 0.008
S6	625	23.53	2.845 ± 0.012

**Figure 5:**
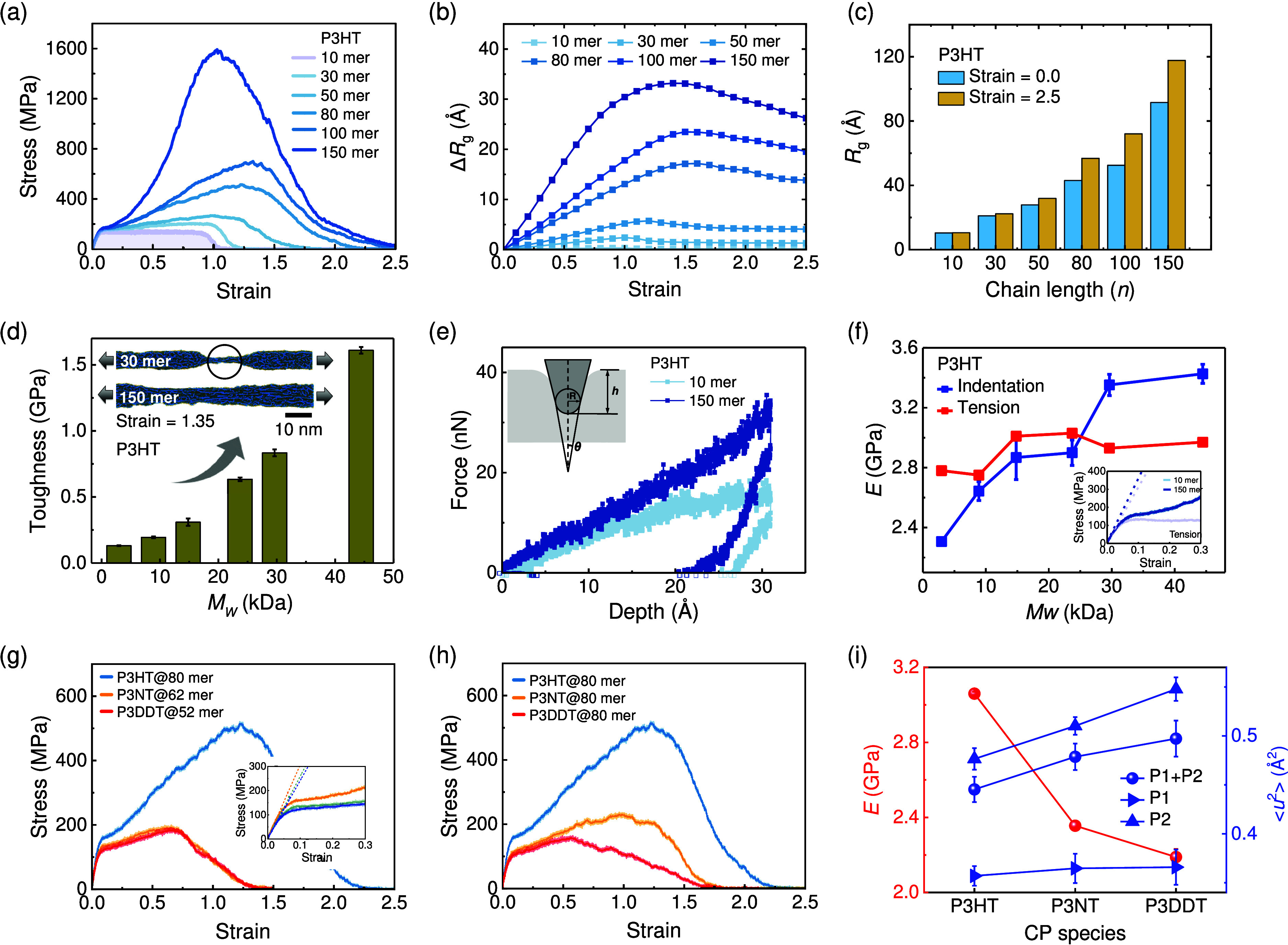
Mechanical and conformational property characterization of P3AT thin films with different *M*_*w*_ and side-chain lengths. (a) Stress–strain curves of the P3HT thin film with different *M*_*w*_ (or *n*). (b) The change of radius of gyration (Δ*R*_*g*_) as a function of strain and (c) the averaged *R*_*g*_ value before and after stretching to 2.5 for P3HT thin films with different *M*_*w*_ (or *n*). (d) Toughness (area under tensile stress curve to 250%) of P3HT with different *M*_*w*_ (or *n*) with the inset showing a snapshot of stretched P3HT thin films with different chain lengths at 135% strain. (e) Representative indentation curves of force vs depth for P3HT thin film systems with 10 and 150 monomers per chain and with the inset showing the nanoindentation theoretical model. (f) Young’s modulus of the P3HT thin film with different *M*_*w*_ obtained from the tensile and nanoindentation test, where *E* from the tensile test is obtained by calculating the slope of the linear fitting of the elastic stress–strain stages, as illustrated in the inset, and *E* from nanoindentation is calculated by using the theoretical model (eq 11). Stress–strain curves of P3AT thin films with (g) the same *M*_*w*_ and (h) the same chain length *n*, respectively. (i) Tensile modulus (*E*) and Debye–Waller factor ⟨*u*^2^⟩ of interior region variation under different side-chain lengths of P3HT (80 mer), P3NT (62 mer), and P3DDT (52 mer) thin films, respectively, where the variation of dynamics of the backbone and side chain are depicted separately.

To characterize the mechanical properties of thin films of P3ATs with varying *M*_*w*_ and side-chain lengths, we use the P3AT thin film systems shown in Table 1 and conduct the uniaxial tension and nanoindentation tests separately. Figure 5a illustrates the stress–strain curves of the P3HT thin film under the effect of *M*_*w*_, revealing that a higher *M*_*w*_ (corresponding to a longer polymer chain) results in elevated stress levels before fracture. As stretching progresses, the radius of gyration (*R*_*g*_) of P3HT chains exhibits a trend similar to that of stress (Figure 5b), and *R*_*g*_ undergoes a decline upon fracture of the film. Figure 5c demonstrates that a higher *M*_*w*_ leads to larger increments in *R*_*g*_ before and after stretching. Note that the entanglement chain length (*N*_*e*_) of P3HT is about 50 repeat units.^55^ The inset of Figure 5d shows the snapshots of a stretched P3HT thin film (strain = 135%) with a chain length below (∼0.6*N_*e*_) and above (∼3*N_*e*_) the entanglement chain length. The low-*M*_*w*_ system becomes fractured via chain pullout (the black circle in the inset of Figure 5d). P3HT thin films with larger *M*_*w*_ values allow more conformational changes and segmental rearrangements within the polymer chains to accommodate the applied strain, preventing fracture initiation. In addition, the area under the stress–strain curve indicates the material’s toughness, which signals the material’s ability to absorb energy under external stress or deformation without fracturing or breaking. Figure 5d clearly shows that increasing the *M*_*w*_ enhances ductility and toughness, which are critically important in flexible devices.

We next study the nanoindentation behavior of P3AT thin film systems, and the representative force (*F*) vs indentation depth (*h*) curve is shown in Figure 5e, revealing stiffening behavior with increasing *M*_*w*_ of the P3HT thin film. A measure of Young’s modulus (*E*) can be derived using the nanoindentation theoretical model^56^ as illustrated in the inset of Figure 5e:
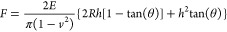
11where *F* is the force collected on the indenter, *h* is the indentation depth, *v* is the Poisson ratio of P3AT (*v* ≈ 0.37) and obtained from the uniaxial tension deformation of the P3HT bulk system (Figure S5 in Supporting Information), which is slightly larger than previous theoretical calculation and simulation results (*v* ≈ 0.35),^22,57,58^*R* is the radius of curvature of the tip apex, i.e., 2 nm in this work, and θ is the half opening angle of the tip. Equation 11 has been successfully implemented to quantify the Young’s modulus of conjugated polymer complex thin film of PEDOT:PSS.^56^ It is also noted that the modulus of the P3HT thin film is not very sensitive to a change in the value of the Poisson ratio.^57^ Results show that *E* obtained from indentation is sensitive to the *M*_*w*_. In contrast, *E* from the tensile test shows *M*_*w*_-independent behavior, which could be attributed to the distinctive deformation mechanisms, where chains are more uniformly stretched and locally compressed under uniaxial tension and nanoindentation, respectively.^59^ Next, we separately fixed the *M*_*w*_ and chain length (*n*) to explore the mechanical properties of the P3AT thin film, where the total number of beads in each system is consistent. Figure 5g and h reveal that regardless of the *M*_*w*_ and side-chain length, the P3HT thin film has the highest *E* and toughness among all P3AT thin films, and the *E* trend is not influenced by *M*_*w*_ or side-chain length (Figure S6), which is mainly attributed to the flexible texture of the side chain. Notably, the P3DDT thin film with a longer side-chain length has the lowest toughness (area under the stress–strain curve) among all of the P3AT thin films with the same chain length (*n*). Since the side-chain length is significantly shorter than the *N*_*e*_, the longer side-chain length causes a lower fraction of backbone, which results in lower toughness in the P3DDT thin film and demonstrates the key role of the backbone on toughness. Figure 5i shows the variation of Young’s modulus (*E*) and DWF (⟨*u*^2^⟩) of the interior region of the P3AT thin film with different side-chain lengths, indicating that a longer side-chain length causes a softer mechanical response, which is attributed to the lower stiffness in the P3AT thin film with a longer side-chain length and consistent with previous work demonstrating the power-law relation between local stiffness and Young’s modulus.^24^ Additionally, the respective ⟨*u*^2^⟩ of P1 and P2 beads in the interior region of the P3AT thin film shows the same trend (Figure 5i).

**Figure 6:**
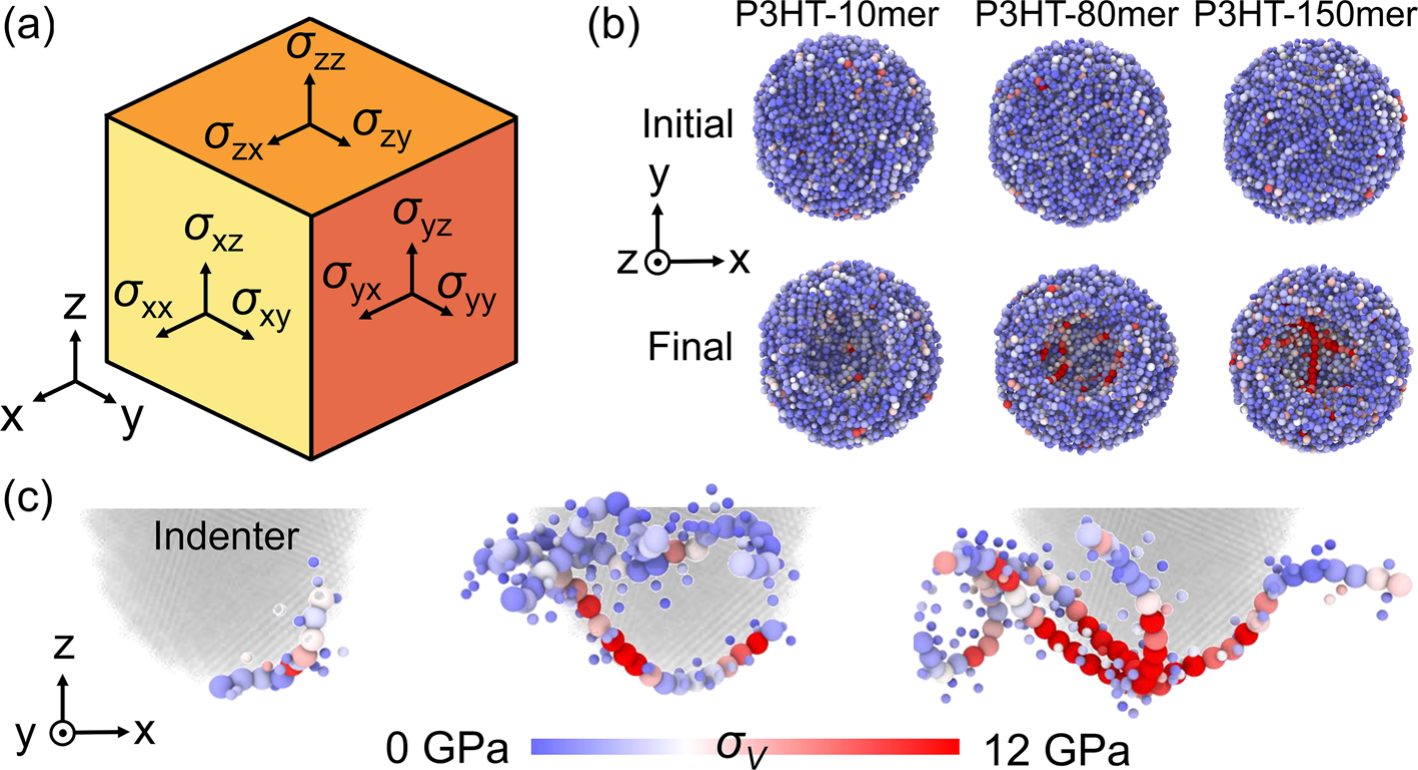
(a) Six stress components, i.e., σ_*xx*_, σ_*yy*_, σ_*zz*_, σ_*xy*_ (= σ_*yx*_), σ_*xz*_ (= σ_*zx*_), and σ_*yz*_ (= σ_*zy*_), to determine the Von Mises stress of each bead. (b) Von Mises stress, σ_*v*_, distribution of P3HT thin film systems with chain lengths of 10, 80, and 150 monomers before and after nanoindentation with a final depth of 30 Å. Only polymers near the indenter are shown for clarity. (c) σ_*v*_ distribution of the selected P3HT chain that contains the highest σ_*v*_ and is attached to the bottom surface of the indenter in P3HT thin film systems with chain lengths of 10, 80, and 150 monomers. The color bar indicates the σ_*v*_ gradient for panels (b) and (c).

To gain insights into the mechanism of nanoindentation, we calculate the Von Mises stress (Figure 6a), σ_*v*_, for the CG bead near the indenter, where the red and blue colors denote the high and low σ_*v*_ values. Figure 6b shows the initial and final snapshots of the local region of P3HT thin film systems with 10, 80, and 150 monomers per chain, respectively. P3HT systems with longer chain lengths possess higher σ_*v*_ values at the bottom of the pit formed by nanoindentation, explaining the high *F* and *E* values obtained from the indentation test. Furthermore, the P3HT chain containing the highest σ_*v*_ in each system is shown in Figure 6c. For the P3HT thin film with lower *M*_*w*_, chains tend to be more easily displaced during the nanoindentation, causing local stress dissipation. Conversely, the long chains experience different internal strains due to more constraints under indentation deformation,^60^ which causes high heterogeneity in σ_*v*_ for the chain surrounding the indenter. As a result, the nanoindenation tests show stronger *M_w_*-dependent behavior as compared to the tensile tests. Additionally, we characterize the chain conformation of the thin film before and after nanoindentation in Figure S11 in the Supporting Information.

### Chain Conformation

**Figure 7:**
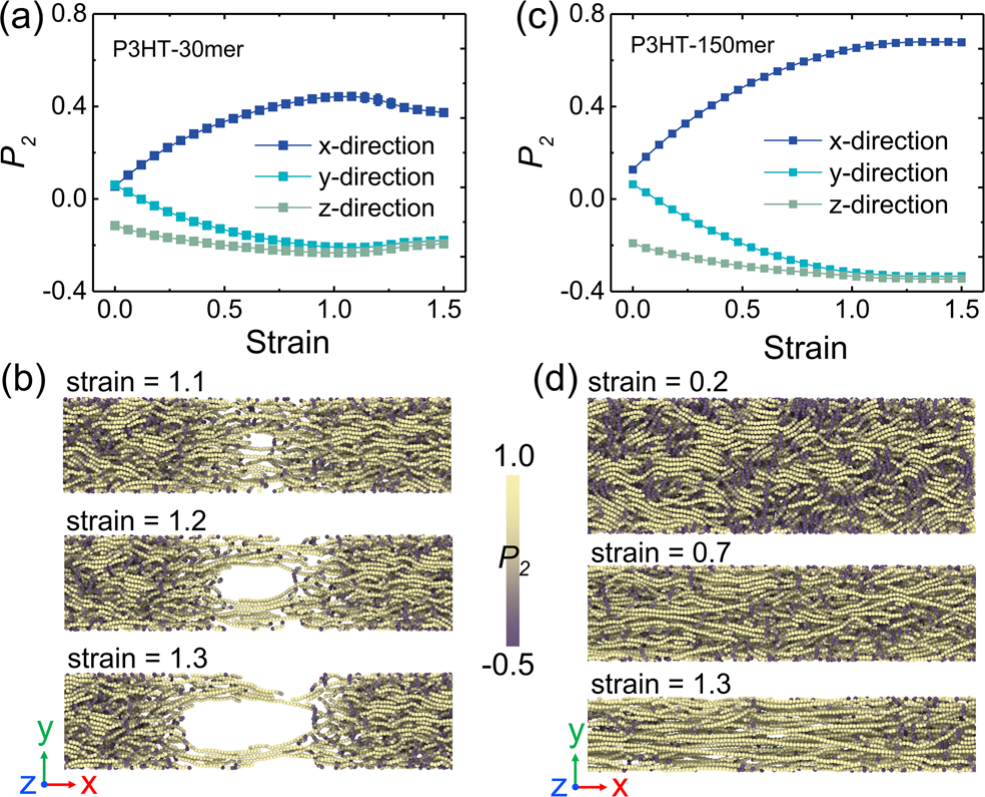
Backbone orientation in three directions of the system during the uniaxial tension deformation of P3HT thin films with (a) 30 and (c) 150 monomers per chain. (b) Top view snapshots of the P3HT thin film (30 mer) at strains of 1.1, 1.2, and 1.3, respectively, where the crack occurs by chain pullout accompanied by a drop of orientation along the tensile direction when straining ≥ 1.1. (d) Top view snapshots of P3HT thin film (150 mer) at strains 0.2, 0.7, and 1.3, respectively, where polymer chains continuously align in the stretching direction and the film gradually narrows. Only backbones are shown here for clarity, and chains are colored using their local ordering parameter *P*_2_.

During stretching, the polymer chains tend to align in the deformation direction. Herein, we assess the backbone orientation parameter of *P*_2_ (eq 9) for P3HT thin films with *M*_*w*_ values of 8.890 and 44.448 kDa. Specifically, *P*_2_ = 1 and −0.5 signifies that all backbone bonds are perfectly parallel and perpendicular to the stretching direction, respectively. *P*_2_ = 0 denotes the randomly oriented backbone bonds in the system, i.e., the pure amorphous region. Figure 7a,c show that, initially, the backbone bonds are randomly oriented in the *x*- and *y*-directions. The presence of a free surface may cause partial alignment of the backbone bonds perpendicular to the thin film plane in the early stages, as shown in Figure S8. As stretching progresses, the backbone gradually aligns with the tensile direction, which will impact the charge transport and improve the electronic properties of the thin film.^13,61^ For the P3HT thin film with a small chain length (∼0.6*N_*e*_), the crazing fiber is formed during the tensile deformation (Figure 7b) and then fractured, while the P3HT film with a long chain length (∼3*N*_*e*_) becomes thinner and narrower upon stretching; that is, it shows necking (inset of Figure 5d and Figure 7d). Note that the drop in the chain orientation parameter could serve as an indicator of the thin film fracture at a strain of 1.1 (Figure 7a,b). Additionally, the *P*_2_ value of a high-*M*_*w*_ thin film (Figure 7c) is much larger than that of a low-*M*_*w*_ thin film (Figure 7a) at the final stage because of sufficient sliding between long chains. As for the side-chain alignment, we characterize the P1–P2 and P2–P2 bond vectors in the P3HT thin film system with 150 monomers per chain during uniaxial tension deformation. Figure S7 compares the backbone and side-chain orientations, demonstrating that the backbone is oriented more than the side chains due to the constraint of the adjacent P1 bead and short side-chain length compared to the backbone. Moreover, we analyze the RDF of P3HT (80 mer), P3NT (62 mer), and P3DDT (52 mer) in the equilibrated state to explore the structural property of the CP thin film, as shown in Figure S9 in the Supporting Information.

**Figure 8:**
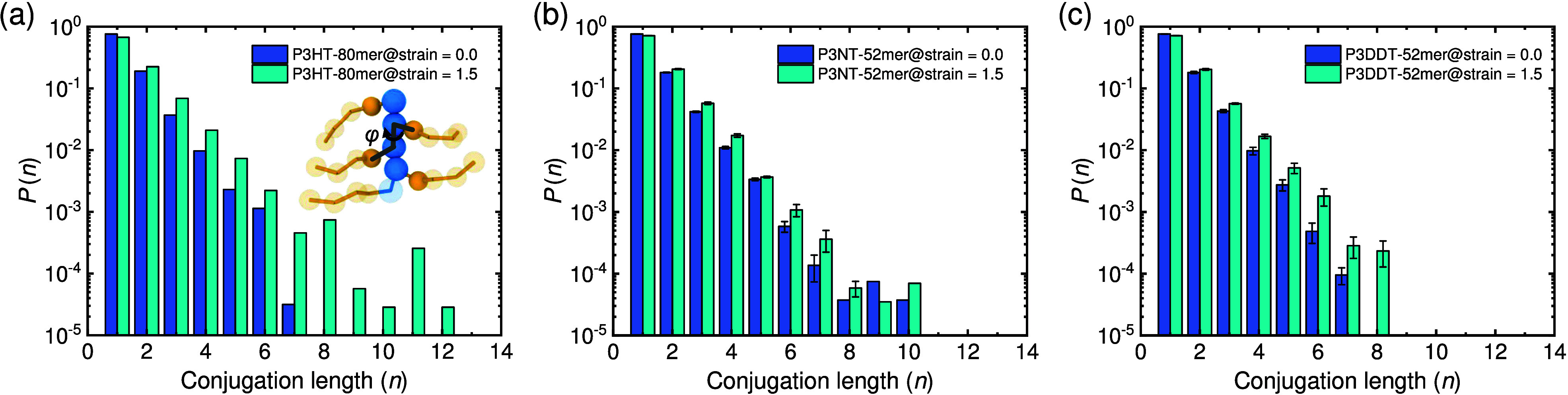
Probability distribution of conjugation length (*n*) in the (a) P3HT (80 mer), (b) P3NT (62 mer), and (c) P3DDT (52 mer) thin film systems at strains of 0.0 and 1.5, where stretching induces longer conjugation length and higher probability. The error bars are obtained from three independent runs.

Next, we introduce conjugation length (CL) as a simple estimation for assessing the electronic properties of CPs under deformation. We chose a P3HT thin film system with 80 monomers per chain as the model system. CPs are organic macromolecules characterized by a backbone chain consisting of alternating double (aromatic) and single bonds; that is, their overlapping π-orbitals enable a system of delocalized π-electrons, resulting in fascinating electronic and optical properties. However, the bends of the backbone and rotations of inter-ring bonds are two major factors causing chain torsions that deviate from planarity, which destroy the extent of the π-conjugation and break the CP backbone into electronically isolated units. From previous work,^62^ we can measure the intermonomer dihedral angle to estimate the CL (inset of Figure 8a). Namely, the P3AT chain segments are considered conjugated if the intermonomer dihedral angle falls within a specific threshold value. Zhang et al.^63^ proved that the excitation energies show a slight change (0.24 eV) within the 40° intermonomer dihedral relatives to that of the planar chain, which is used as the threshold dihedral angle value in this work for calculating CL. Figure 8a reveals the effective CL (ECL, maximum CL in one chain) of 7 monomers (∼2.7 nm) for the P3HT thin film (80 mer) system initially, and the effective CL becomes longer with a higher probability after stretching to 150%, i.e., 12 monomers (∼4.7 nm), which is similar for P3NT and P3DDT systems (Figure 8b,c). Previous neutron scattering experiments revealed that the effective CL of P3HT at room temperature is 3 nm in dilute dichlorobenzene solutions.^64^ The simulation work from Tsourtou et al. demonstrated the effective CL of 2.4 nm of P3HT (90 mer) at 640 K and decreased effective CL with increasing temperature,^65^ which is comparable with our results. The CL analysis for P3HT thin film systems with different *M*_*w*_ values is described in Figure S10, where a high-*M*_*w*_ system has a longer effective CL. Exclusively employing the CG model and geometric parameters to delineate the conjugation status between adjacent thiophene rings yields a reasonable approximation of the ECL. However, achieving a more precise assessment of the maximal extent of conjugation and its interplay with *M*_*w*_ necessitates direct quantum mechanics computations and atomistic modeling.

### Scratching Characterization

Finally, we characterize the scratch behavior of the P3AT thin film with different *M*_*w*_ and side-chain lengths. The scratch performance of conjugated polymer films is important in evaluating their mechanical durability and resistance to surface damage, aiding in understanding the film’s ability to withstand abrasion or scratching forces, which is crucial for applications requiring robustness, such as protective coatings, electronic devices, or optoelectronic materials.^66^ Herein, we calculated the instantaneous friction coefficient of each system using the ratio between the friction force and normal force.^67^
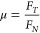
12

**Figure 9:**
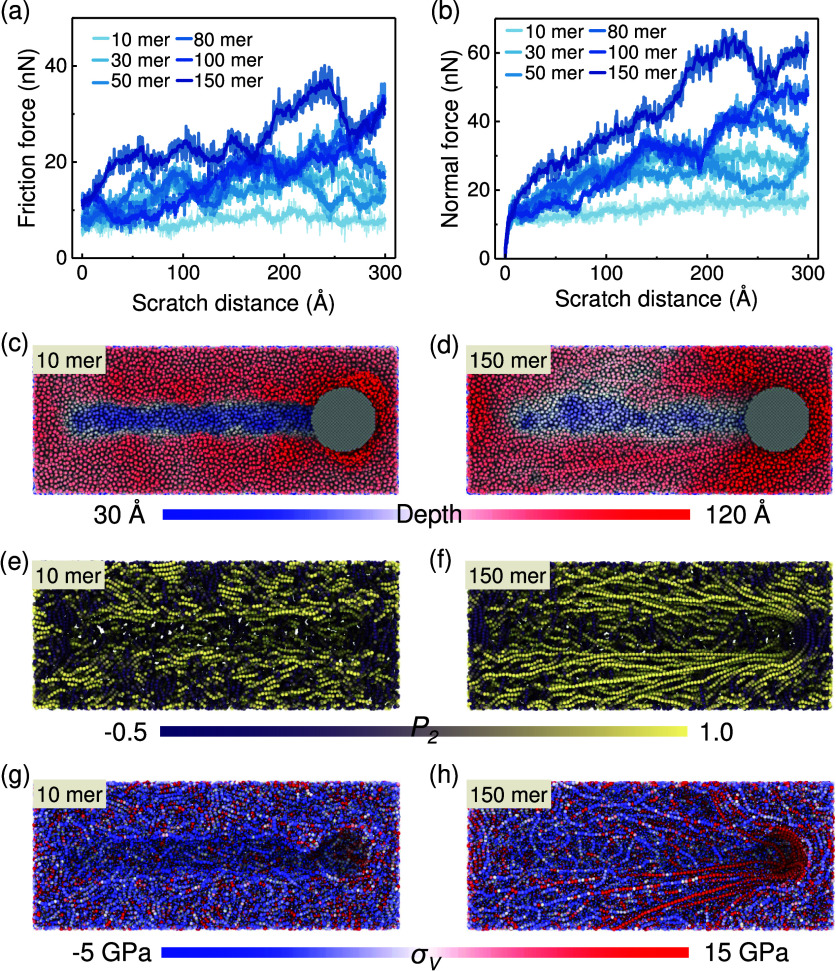
Scratch characterization of P3HT thin films with different *M*_*w*_. (a) Friction force and (b) normal force as a function of the scratch distance for the P3HT thin film with different *M*_*w*_; the error bands are calculated from three independent runs. (c, d) Snapshots of the scratch process of P3HT with 10 monomers per chain for scratch distances of 0 and 300 Å, respectively, with the color bar showing the depth vertical to the thin film. (e, f) Snapshots showing the top view of the backbone of the P3HT thin film (150 monomers per chain) during scratching at distances of 0 and 300 Å, respectively. Chains are colored according to their local alignment *P*_2_ = ⟨(3 cos^2^ θ – 1)/2⟩. (g, h) Von Mises stress distribution of atoms for the P3HT thin film (150 monomers per chain) during the scratching process at scratch distances of 0 and 300 Å, with the color bar below denoting the atomic Von Mises stress.

Figure 9a,b represents that as the scratch testing proceeds, the friction and normal force of the P3HT film with high *M*_*w*_ gradually increase, primarily due to the accumulation of more chains by the indenter and sufficient chain friction, as shown in Figure 9f,h. The average friction coefficient, μ, is determined during the scratch distance from 100 to 300 Å. Results show no significant difference in the friction coefficient for all systems, as shown in Figure S12a,b. It is noted that the friction coefficient shows a slight increase with increasing scratching depth (Figure S12c). The scratch test of P3AT with different *M*_*w*_ and side-chain lengths is described in Figures S12 and S13. Unlike the scratch test of metal materials,^68^ the P3HT thin film exhibits an uneven scratch path (Figure 9c,d) especially for high-*M*_*w*_ systems due to the flexible nature of the polymer, and polymer chains recover the scratch path after scratching. Local chain orientation analysis of the scratch shows highly aligned chains on both sides of the scratch path (Figure 9e,f) for P3HT with a large *M*_*w*_. Additionally, increasing the *M*_*w*_ of P3HT causes more pronounced chain alignment along the scratch path (Figure S13) because of sufficient chain sliding. As for the P3AT thin film system with different side-chain lengths, the P3DDT thin film shows a less pronounced chain orientation due to the low fraction of backbone in the system (Figure S12). Figure 9g,h shows the Von Mises stress of each bead during the scratching process, and the results show the heterogeneous distribution of σ_*v*_ in the thin film. The polymer chain exhibits a high σ_*v*_ as a result of chain sliding when the indenter captures the middle portion of the chain, contributing to the high friction and normal forces during scratching. Notably, the friction coefficient is influenced by the type of indenter, shape of the indenter, indenter radii, scratching velocity, surface roughness, initial indentation depth, temperature, and so on, which is worth studying in the future, but is currently beyond the scope of our work.^69−71^

## Conclusions

In this work, we successfully implemented our new chemistry-specific coarse-grained molecular dynamics model to systematically investigate and characterize the mechanical and conformational properties of free-standing conjugated polymer thin films through uniaxial tension, nanoindentation, and scratching, where varying *M*_*w*_ and different architectures of P3ATs are considered. With the increase in the side-chain length of P3AT, the dynamics within the film’s internal regions is faster, leading to lower local stiffness, although the effective thickness of the free-standing P3AT thin film is independent of *M*_*w*_ and P3AT side-chain length. Because of this soft layer, thinner films have an overall lower modulus and toughness, as shown by uniaxial tension tests. Interestingly, due to different deformation mechanisms, the nanoindentation test shows a higher elastic modulus in the P3HT thin film with increasing *M*_*w*_, an effect not observed in uniaxial deformation because of the different deformation modes. The high *M*_*w*_ of P3HT also significantly improves the toughness of the thin film. The longer side-chain length of P3AT has a lower Young’s modulus and local molcular stiffness in the thin film systems. A chain orientation analysis shows a stronger backbone alignment in the P3HT thin film with high *M*_*w*_ upon stretching because of sufficient chain sliding. Fracture in low *M*_*w*_ films is accompanied by a reduction in the backbone alignment with the deformation direction. Stretching can also improve the conjugation length, which is beneficial for the electric properties of the thin film. Additionally, the scratching test indicates high friction and normal forces in a thin film system with high *M*_*w*_, while no significant difference in friction coefficient is observed for all P3AT thin film systems regardless of thickness and polymer side chain. This work systematically explored the important role of *M*_*w*_ and side-chain length of P3AT on the mechanical and conformational properties of conjugated polymer thin films, providing insight into tuning the properties of new flexible electronics.

## Supporting Information Available

AA and CG simulation; thin film characterization (Figures S1–S3); radius of gyration change vs strain (Figure S4); mechanical property of the thin film (Figures S5, S6); chain conformation upon stretching and scratching (Figures S7–S13); potentials of P3AT CG models (Table S1); ER function and parameters (Table S2) (PDF)
