# Supplementary material for: A Molecular Dynamics Study of Mechanical and Conformational Properties of Conjugated Polymer Thin Films
Source: Macromolecules. 2024 May 20;57(11):5130–42. doi: 10.1021/acs.macromol.4c00232 (PMC11171455; doi:10.1021/acs.macromol.4c00232)
Supplement: Supplementary file 1 — ma4c00232_si_001.pdf [file ma4c00232_si_001.pdf]

Supporting Information for A molecular dynamics study of mechanical and conformational properties of conjugated polymer thin films

Yang Wang,[†,‡] Zhaofan Li,[¶] Kangmin Niu,[*,‡] Wenjie Xia,[*,¶] and Andrea Giuntoli[*,†]

†*Zernike Institute for Advanced Materials, University of Groningen, 9747 AG, Groningen, The Netherlands*

‡*School of Materials Science and Engineering, University of Science and Technology Beijing, Beijing 100083, China*

¶*Department of Aerospace Engineering, Iowa State University, Ames, IA 50011, United States*

E-mail: niukm@ustb.edu.cn; wxia@iastate.edu; a.giuntoli@rug.nl

AA and CG simulation

In this work, we get the CG bonded potentials of P3ATs using the average distribution functions from all-atomistic models of P3HT, P3NT, and P3DDT, which is slightly different from our previous work since the non-dominant nature of bonded interactions in influencing the mechanical performance.[1] The atomistic simulation details of P3ATs are well elaborated in our previous work. Initially, we built the AA models of P3HT, P3NT, and P3DDT with 30 monomers per chain using the Materials Studio platform. Then we assign the model with the Dreiding force field[2] using the pysimm (a python package).[3] The Dreiding force field was previously used to study the self-organization and structure of the P3HT chain in pure crystalline and pure amorphous phases, respectively.[4] The Gasteiger method[5] is adopted to calculate system atomic charges for electrostatic interactions. Energy minimization is first performed using the conjugate gradient algorithm.[6] Next, the system is equilibrated in the melt state at a high T of 1000 K under the NPT ensemble for 2.5 ns with the pressure ramping from the initial 1000 atm to the final 100 atm. Afterward, the system is further cooled to 300 K at 1 atm pressure under an NPT ensemble for 4 ns before sampling. Following equilibration, a 2 ns dynamics run under an NPT ensemble is performed to collect the atomistic trajectories with a sampling interval of 1 ps. The same simulation procedure is performed for P3HT, P3NT, and P3DDT, respectively, to get the average bonded distributions of bond, angle, and dihedrals as the target distributions. The simulation details are the same for the AA and CG systems to get the bonded probability distributions except for the timesteps of 1 and 4 fs are respectively employed. The larger timesteps used in the CG representation are due to the smoother potential energy landscape compared to the AA counterpart. After 5-7 rounds of iterative Boltzmann inversion, the bonded probability distribution functions of the CG model are consistent with the AA model, and the representative CG bonded potentials are shown in **Figure 2** in the main text. The bonded potentials and parameters of the CG model are listed in **Table S1**.

As for the nonbonded interactions, we use a liquid monomer bulk system with 800 P3DDT monomers to calculate the radial distribution function (RDF) between different CG beads.

Then, using direct Boltzmann inversion and energy-renormalization approach, we can get the temperature- and architecture-transferable nonbonded parameters of the Lennard Jones potential, as shown in **Table S2**. The detailed information on energy renormalization for characterizing the nonbonded interactions is illustrated in our previous work.[7] Using the ER-corrected P3AT CG model, we can faithfully capture the density, dynamics, and mechanical properties of P3AT over a wide temperature range.

Thin film characterization

We use the Gibbs dividing surface (GDS) method to get the effective thin film thickness based on the density profile. To illustrate the GDS, we chose a P3HT thin film system with 80 monomers per chain. **Figure S1**a shows the density distribution in the z-axis. In the conventional model, the position of the Gibbs dividing surface can be obtained by fitting the density profile using the following functional form[8]

$$\rho(z) = \frac{1}{2}(\rho_i + \rho_v) - \frac{1}{2}(\rho_i - \rho_v)\tanh[(z - z_0)/d_i] \tag{1}$$

where ρ_i and ρ_v represent the interior and vacuum densities, respectively, z_0 is the positions of Gibbs's dividing surface and d_i is the thickness of the interface. **Figure S1**a shows the best-fit curve of the density profile (red line), yielding an effective thickness of 8.12 nm. **Figure S1**b shows the local stiffness distributions of P3HT thin films with different M_w along the z-axis, all systems display a similar local stiffness profile. By averaging the local stiffness of the interior region of all systems, **Figure S1**c depicts the $1/\langle u^2 \rangle$ vs. M_w, where the P3HT system with 10 monomers per chain represents the lowest stiffness and other systems show a small difference.

Due to the distinct difference in density distribution profiles of backbone bead P1 and side-chain bead P2 along the thickness direction (**Figure S1**d), we separately quantify the dynamics of the P1 and P2 beads for P3ATs thin films with different M_w and side-chain length. **Figure S2**a and b show that for both the backbone bead P1 and side-chain bead P2, the surface region

exhibits stronger dynamics compared to the interior region. **Figure S2**c and d reveal that in the same region (surface or interior), the side-chain bead P2 shows stronger dynamics compared to the backbone bead P1. Overall, the conclusion from **Figure 3**c in the main manuscript is correct. Additionally, as shown in **Figure S3**, for the P3ATs thin films with different side-chain lengths and similar M_w, the side-chain bead P2 exhibits slightly faster dynamics (inset) compared to the backbone bead P1 in both surface and interior regions.

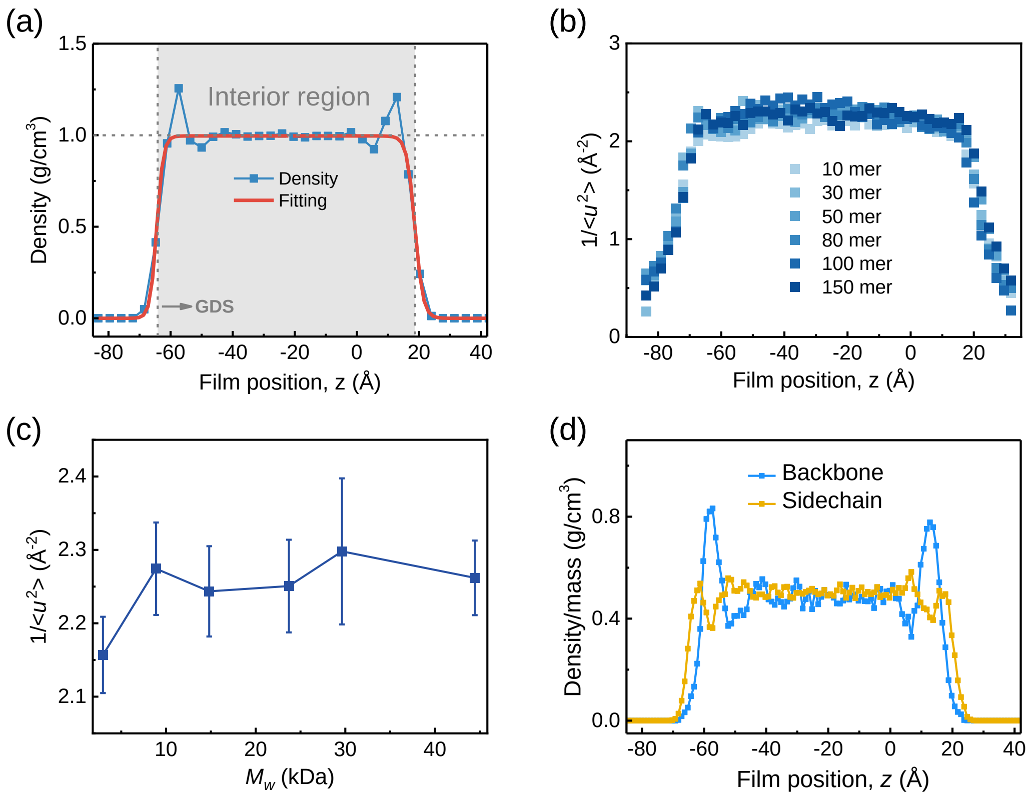

Figure S1. (a) Illustration of Gibbs dividing surface for determining the film effective thickness from density profiles of P3AT thin films. (b) Spatial distribution of the local stiffness, $1/\langle u^2\rangle$, as a function of polymer z position for P3HT thin film systems with different M_w. (c) Average local stiffness of the interior region of P3HT thin film as a function of M_w, where the interior region is determined using the GDS method shown in panel (a). (d) Density profiles of backbone and side-chain bead along the z-axis for the P3HT thin film with a M_w of 2.963 kDa (10 monomers per chain).

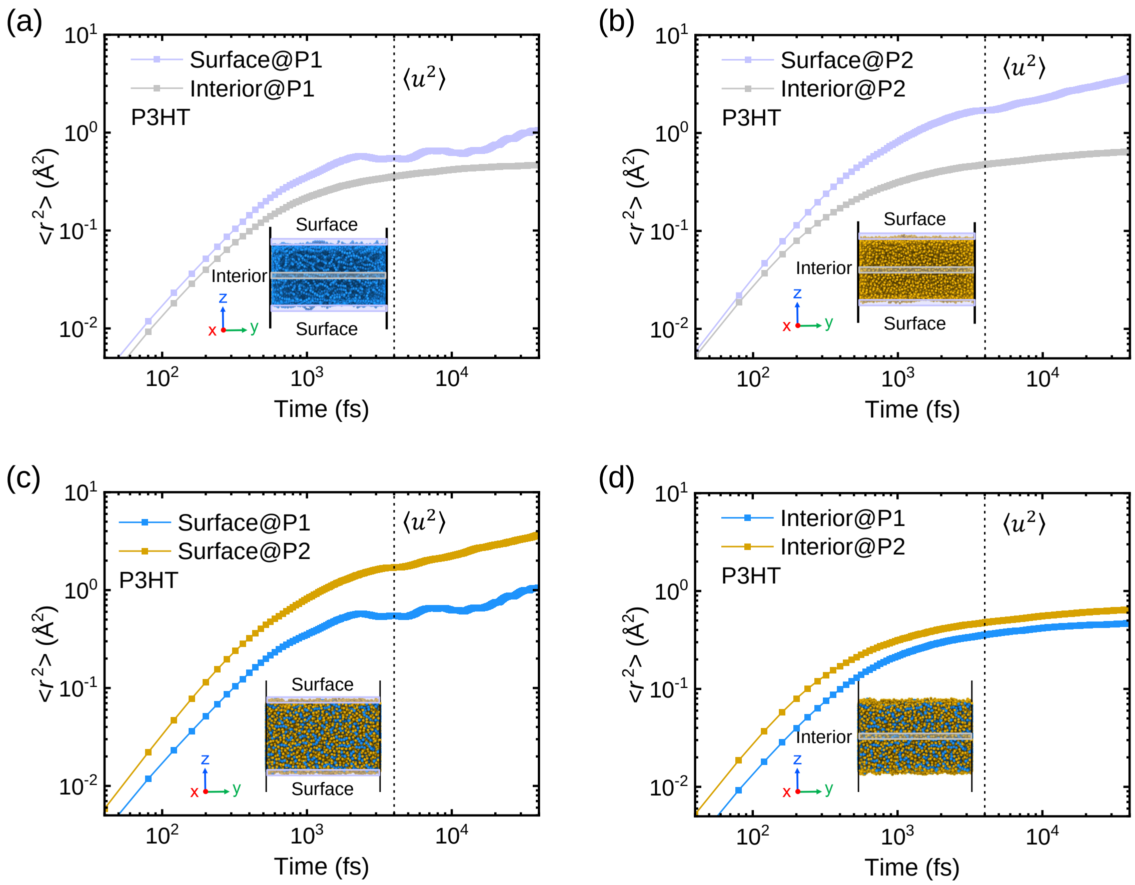

Figure S2. Independent layer-by-layer dynamics of the backbone and side-chain beads of P3HT (80 mer) thin film system. MSD $\langle r^2 \rangle$ vs. time for the interior and surface layer of the (a) backbone and (b) side-chain beads. Comparison of MSD $\langle r^2 \rangle$ vs. time curve for the backbone and side-chain beads in the (c) surface and (d) interior layer.

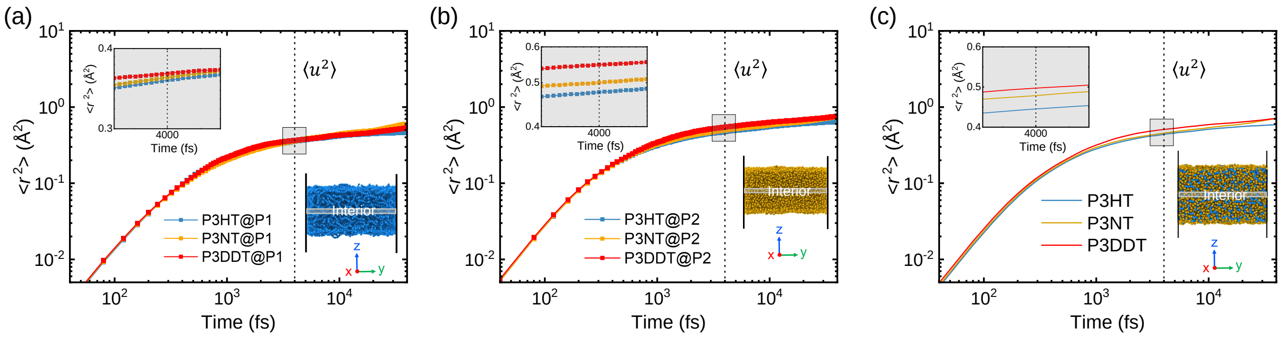

Figure S3. MSD $\langle r^2 \rangle$ vs. time of the (a) backbone bead P1 and (b) side-chain bead P2 in the interior region of P3AT thin films with similar M_w but different side-chain lengths. (c) Overall MSD $\langle r^2 \rangle$ vs. time of the interior region of P3HT (80 mer), P3NT (62 mer), and P3DDT (52 mer) thin film systems, respectively.

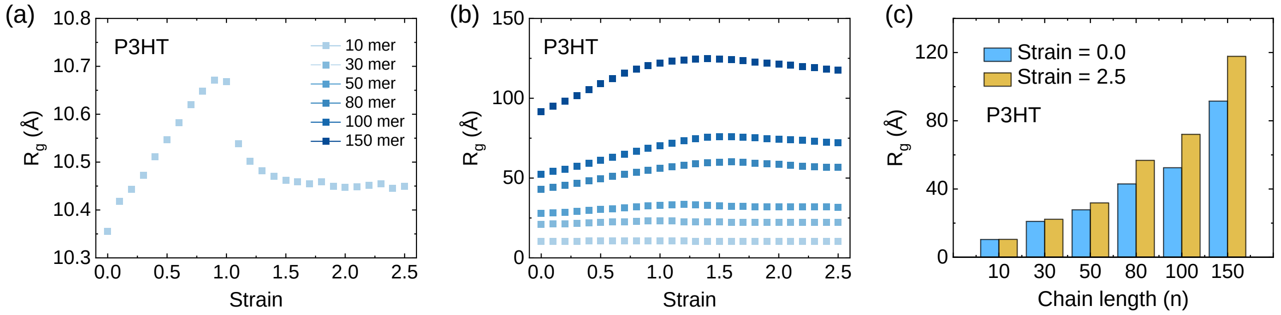

Figure S4. The averaged radius of gyration (R_g) vs. strain of the P3HT thin film with different M_w during the tensile deformation. Panel (a) shows the R_g variation of P3HT thin film with 10 monomers per chain and panel (b) shows the R_g variation of P3HT thin film with different chain lengths. (c) The R_g variation of P3HT thin film with different M_w (chain length, n) before and after stretching to 2.5.

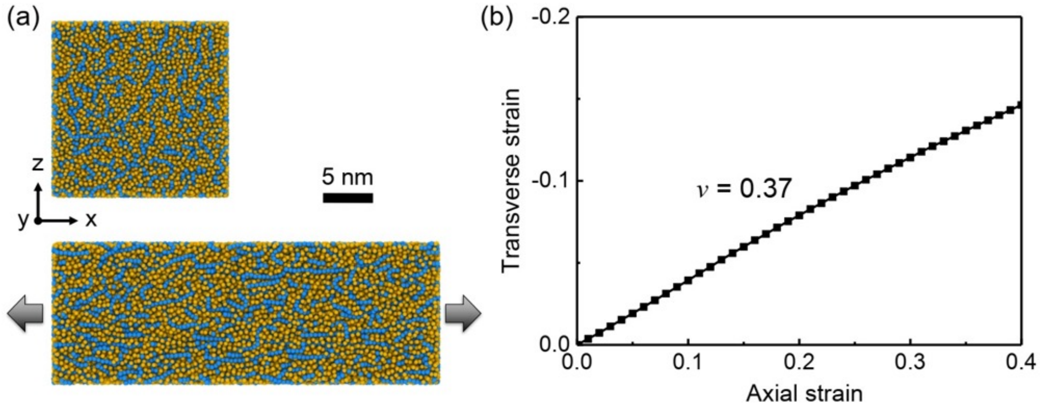

Figure S5. (a) Snapshots of the P3HT bulk system (80 mer) before and after stretching. (b) Transverse strain response to uniaxial loading for P3HT thin film system with the slope denoting the Poisson ratio v.

Mechanical property of the thin film

To get the Poisson ratio of the P3HT model, we build the cubic P3HT bulk system with 250 polymer chains and 80 monomers per chain and then perform the tensile deformation at 300 K. The periodic boundary condition is applied for x, y, and z directions during the equilibration and tensile processes and the tensile strain rate is 0.5 ns^{-1}. **Figure S5**a shows the snapshots of the initial and final states of the bulk P3HT system during the tensile deformation. The relationship between axial and transverse strains during the stretching is depicted in **Figure**

S5b, demonstrating the Poisson ratio of $v = 0.37$, which is slightly larger than the previous works ($v = 0.35$).[9,10]

Figure S6 shows the mechanical properties of P3HT (80 mer), P3NT (62 mer), and P3DDT (52 mer) with similar M_w. It is revealed that P3AT with a short side-chain length has a stronger elastic modulus neglecting the M_w and chain length, which is consistent with previous experimental work that a longer side-chain length of P3AT causes a softer mechanical response.[11] Additionally, P3HT represents the highest toughness (area under the stress-strain curve) compared with P3NT and P3DDT, which is attributed to the longer chain length under the same M_w.

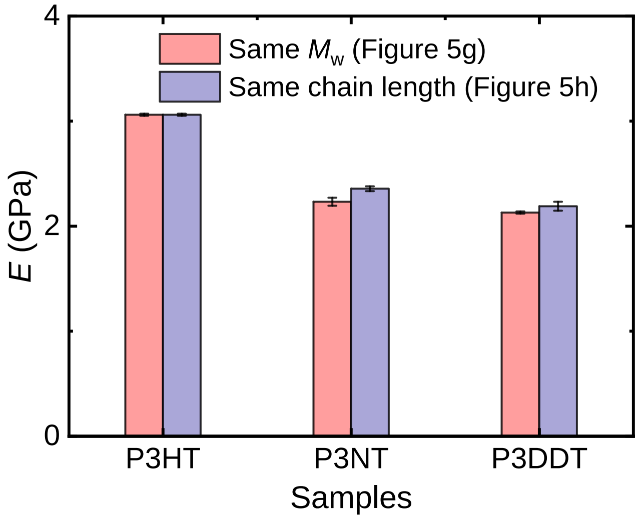

Figure S6. Young's modulus, E, of P3HT, P3NT, and P3DDT thin films with the same M_w and the same chain length, n. E is obtained from the **Figure 5**g and h in the main manuscript.

Chain conformation

The backbone and side-chain orientation evolution of the P3HT thin film system with 150 monomers per chain during the stretching is depicted in **Figure S7**. The orientation of the side chain is weaker compared to the backbone. Additionally, P3HT shows the negative P_2 in the z-direction at the initial state, which we attribute to the thin film structure and surface effect. **Figure S8** demonstrates the layer-by-layer backbone orientation parameter of P_2 of P3HT (150

mer) along x, y, and z directions at the initial state before stretching, revealing that at the initial state, the value of P_2 along the z-direction is below zero, with the minimum value of P_2 occurring at the interface of both sides.

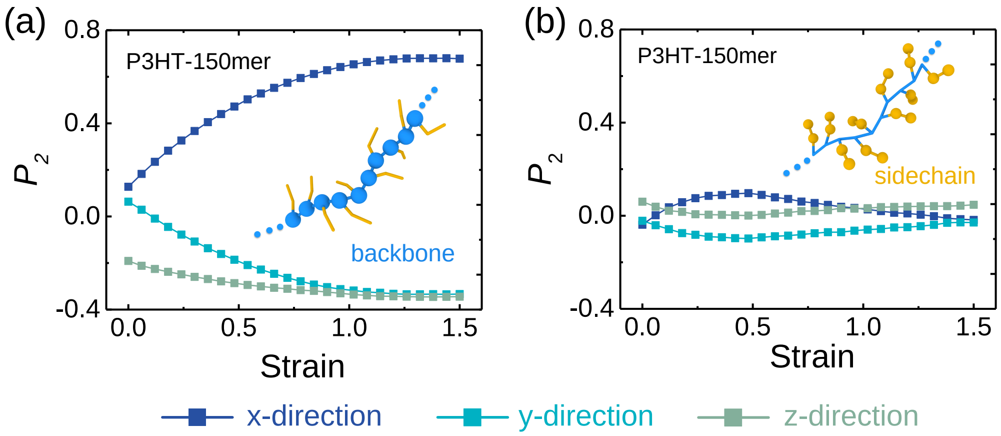

Figure S7. (a) Backbone and (b) side-chain orientation parameter P_2 of P3HT thin film system with 150 monomers per chain during the stretching.

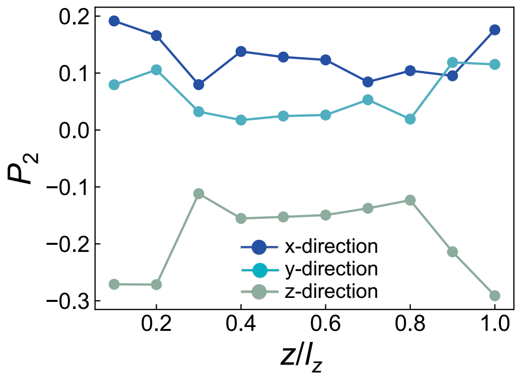

Figure S8. A layer-by-layer distribution of the backbone orientation parameter, P_2, along the thickness direction for the P3HT (150 mer) thin film system at the initial state.

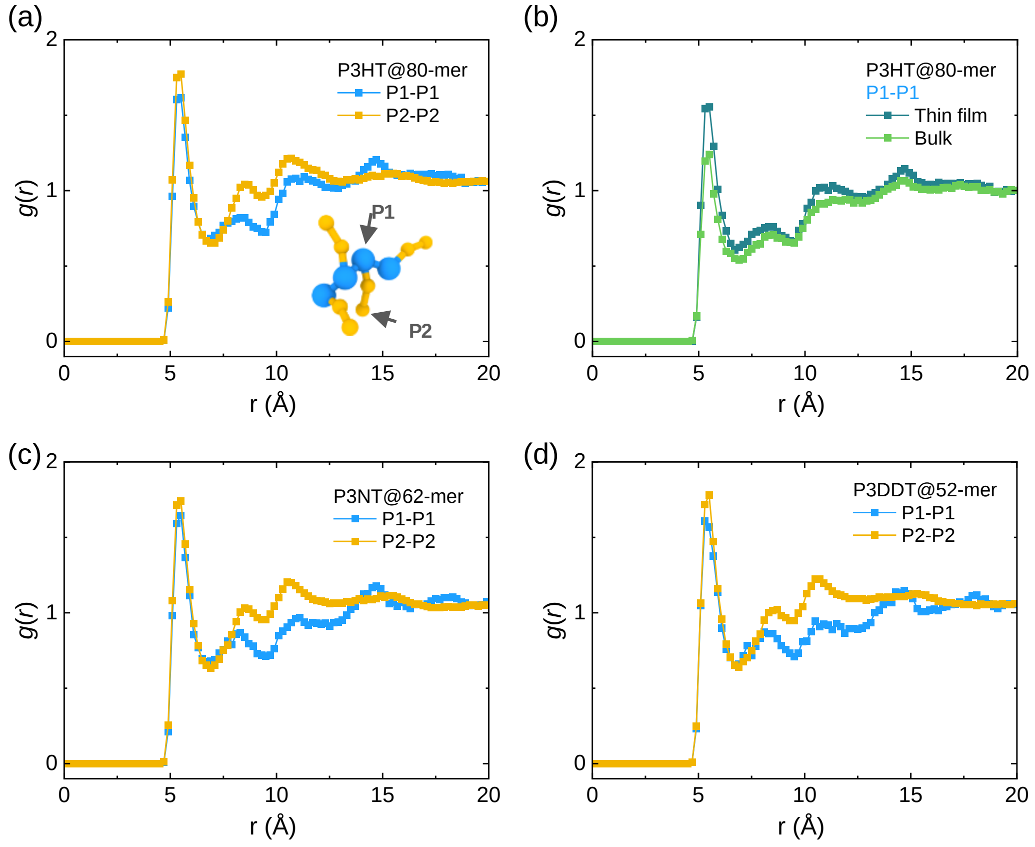

Figure S9. The radial distribution function (RDF) of P1-P1 and P2-P2 for the (a) P3HT, (c) P3NT, and (d) P3DDT CG thin film system, respectively. The RDF of P1-P1 for the P3HT CG bulk system is shown in panel (b) for comparison.

The radial distribution function (RDF) provides information about the spatial distribution of particles around a reference particle and reveals local packing/ordering in disordered systems. The RDF curves of P3HT (80 mer), P3NT (62 mer), and P3DDT (52 mer) with similar M_w are calculated, where CG beads in the same molecule separated by more than three bonds are considered, as shown in **Figure S9**. The RDF of the bulk P3HT system (80 mer, 250 chains) is also shown in **Figure S9**b for comparison. It is revealed that for P3ATs with different side-chain lengths, the side-chain P2 beads are more ordered than P1, as shown by the increased magnitude and sharpness of the first P2-P2 $g(r)$ peaks (**Figure S9**a,c,d), owing to that the side-chain P2 beads have a higher probability of mutual proximity compared with backbone P1 beads, which is consistent with previous CG modeling work.[12] Notably, **Figure S9**b shows that the backbone P1 bead is more ordered in the thin film system (high magnitude of the first peak) compared

to that in the bulk system, which could be attributed to the partial alignment of the backbone near the surface (**Figure S8**).

As for the conjugation length (CL), **Figure S10** shows the effective CL of P3HT thin film systems with different M_w (chain lengths). Results show that stretching displays the effect of raising the effective conjugation length and relative probability, especially for the P3HT thin film with a longer chain length, which is proven beneficial to the charge transport along the conducting channel direction.[13] To explore the effect of the indentation on the conformation change of the P3AT thin film, we choose the P3HT (80 mer) thin film as an example and characterize the conformation behavior of the thin film. **Figure S11**a shows that when the indenter is gradually removed, most of the pitted parts of the film are recovered (middle and right panels of **Figure S11**). We next characterize the RDF of the P3HT thin film before and after indenting, revealing that the indentation test significantly enhanced the local ordering of the film as the beads near the indentation region were compressed more tightly**Figure S11**b and c. However, the indentation test destroys the conjugation length as CL decreases as indenting to 30 Å, as shown in **Figure S11**d.

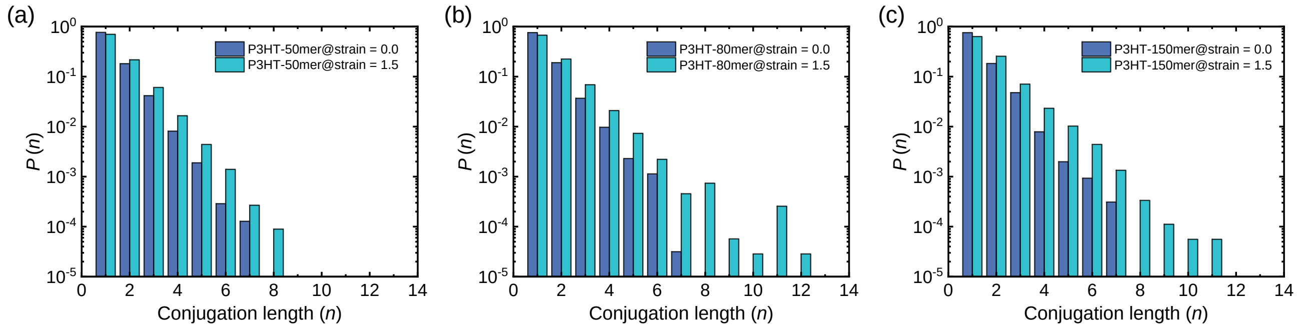

Figure S10. Conjugation length probability distribution before and after the stretch to the strain of 1.5 for the P3HT thin film system with (a) 50, (b) 100, and (c) 150 monomers per chain.

Figure S12a reveals the insignificant difference in friction coefficient for P3AT thin film systems with different side-chain lengths. However, the chain alignment is more evident in the P3NT system compared with P3DDT after scratching. **Figure S13** shows the local chain alignment parameter, P_2, of P3HT thin films with different M_w (or chain lengths). Due to the

chain sliding, the more pronounced aligned chains are detected in the system with a higher M_w after scratching. To test the effect of the scratching depth on the friction behavior, we performed the scratching test of P3HT thin film with chain lengths of 10 and 150 monomers. It is worth noting that as the scratch depth increases, the friction coefficient increases slightly because the increment of the friction force (F_T) is larger than the increment of the normal force F_N, as shown in **Figure S12**c, which could be attributed to that for the larger scratching depth inducing more resistance to the movement of the indenter.

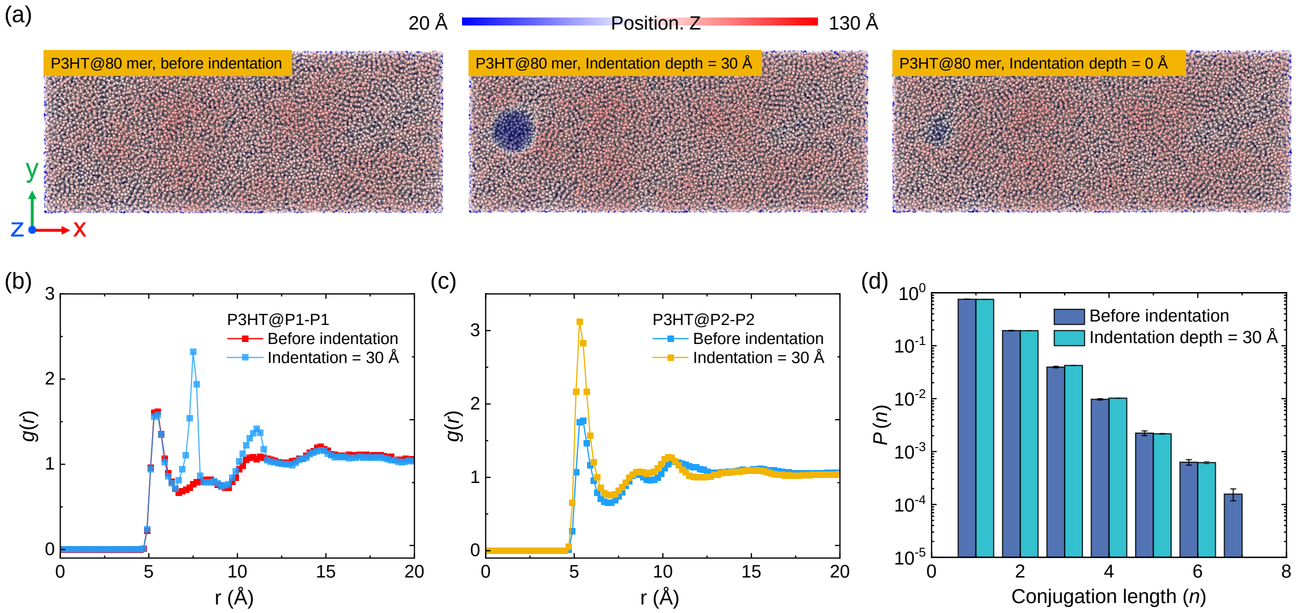

Figure S11. (a) Snapshots of the top views of P3HT (80 mer) thin film before nano-indentation (left), at an indentation depth of 30 Å (middle), and after removing the indenter (right). RDF curves for the (b) backbone beads P1 and (c) side-chain beads P2 in the P3HT (80 mer) thin film before and after indenting at a depth of 30 Å. (d) The conjugation length distribution of P3HT thin film (80 mer) before and after nanoindentation with an indent depth of 30 Å.

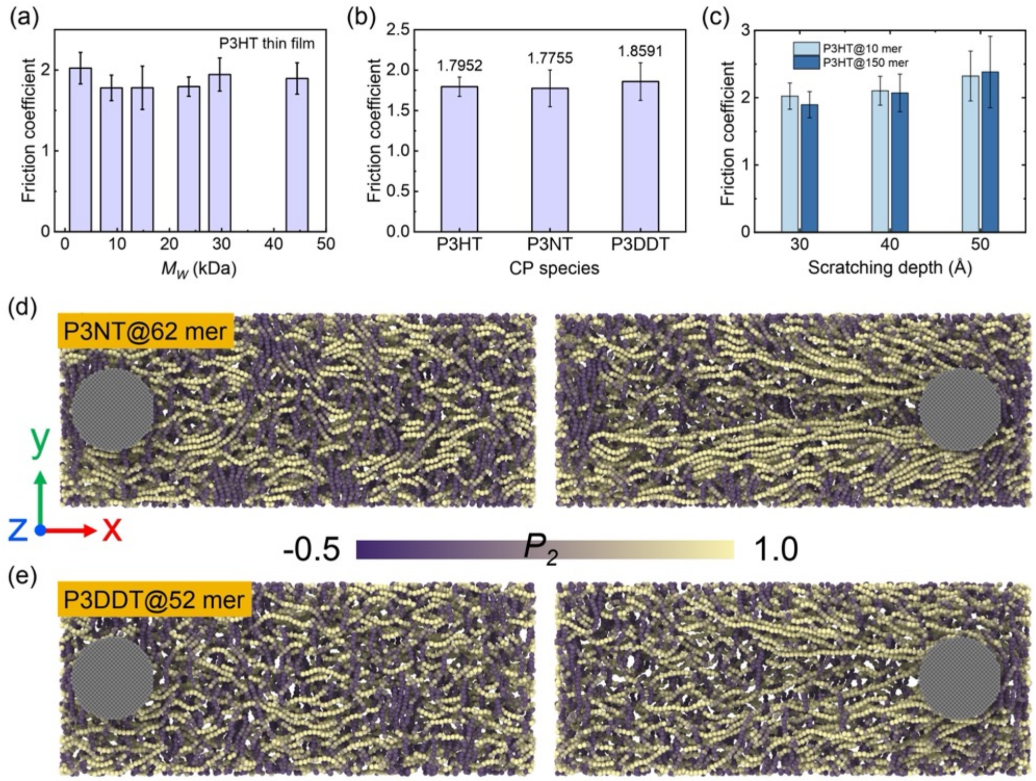

Figure S12. (a) The friction coefficient of P3HT thin films with different M_w. (b) The friction coefficient of P3AT thin films with similar M_w and different side-chain lengths, *i.e.*, P3HT (80 mer), P3NT (62 mer), and P3DDT (52 mer) thin film. (c) The friction coefficient of P3HT thin films with chain lengths of 10 and 150 and different scratching depth. The friction coefficient of the thin film is derived by averaging the ratio between friction and normal force over a range of scratch distances, specifically from 100 to 300 Å.

(d,e) Top view snapshots showing the initial and final state of scratching for the P3NT and P3DDT thin films. Chains are colored according to their local chain alignment $P_2 = \langle(3cos^2\theta - 1)/2\rangle$.

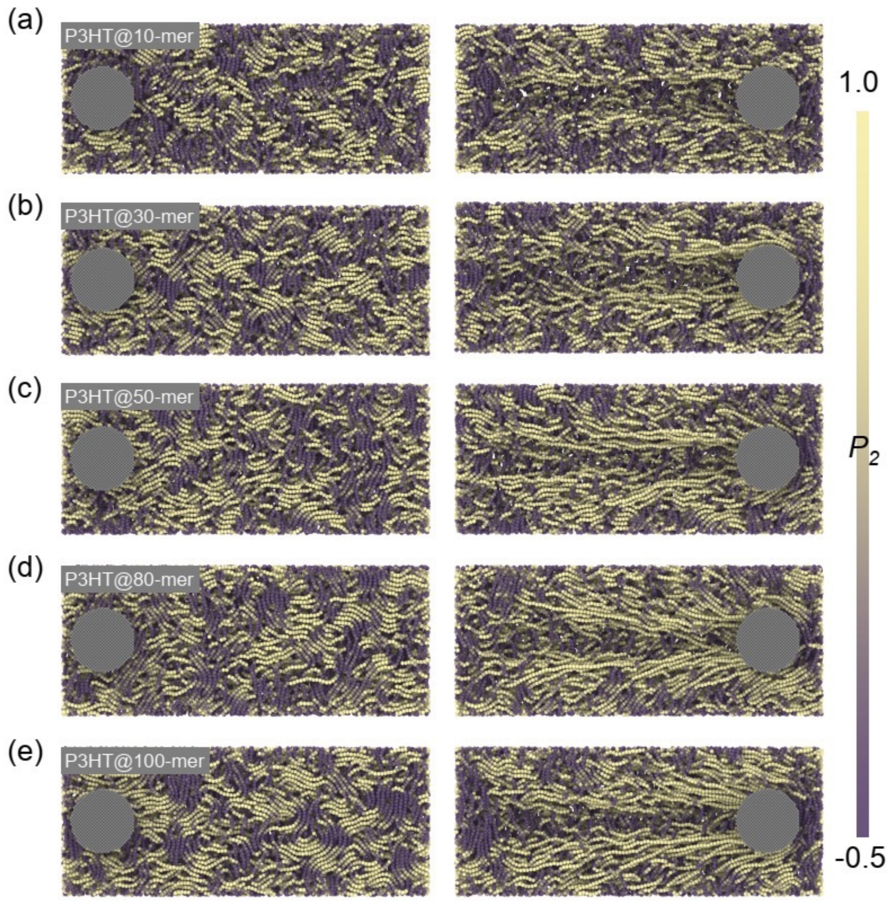

Figure S13. Top view snapshots showing the initial and final state of scratching for the P3HT thin film with different chain lengths. Chains are colored according to their local alignment $P_2 = \langle (3cos^2\theta - 1)/2 \rangle$. For the P3HT thin film with a longer chain length, the chain alignment is more apparent than that with a short chain length.

Table S1. Functional Forms and Parameters of Bond, Angle, Dihedral, and Improper Interactions for CG Model of P3AT conjugated polymers.

Interaction	Potential form	Parameters
P1-P1 bond	$U_{\mathrm{bond}}(l) = \sum_{i=2}^{4} k_i (l - l_0)^i$	$k_2 = 90.491$ kcal/mol $\cdot$ Å^2, $k_3 = 594.50$ kcal/mol $\cdot$ Å^3, $k_4 = 1951.5$ kcal/mol $\cdot$ Å^4, $l_0 = 3.889$ Å
P1-P2 bond		$k_2 = 64.670$ kcal/mol $\cdot$ Å^2, $k_3 = 222.21$ kcal/mol $\cdot$ Å^3, $k_4 = 201.30$ kcal/mol $\cdot$ Å^4, $l_0 = 4.110$ Å
P2-P2 bond		$k_2 = 92.722$ kcal/mol $\cdot$ Å^2, $k_3 = 595.25$ kcal/mol $\cdot$ Å^3, $k_4 = 949.56$ kcal/mol $\cdot$ Å^4, $l_0 = 3.946$ Å
P1-P1-P1 angle<br>P1-P1-P2 angle<br>P1-P2-P2 angle<br>P2-P2-P2 angle	$U_{\mathrm{angle}}(\theta) = -k_B T \ln \left\{ \sum_i^n \left[a_i \cdot \exp\left(\frac{-(\theta-\theta_0)^2}{b_i} \right) \right] \right\}$	$n = 3$, Tabulated form
P1-P1-P1-P1 dihedral	$U_{\mathrm{dihedral}}(\phi) = \sum_{i=1}^{5} \left[a_i \cdot \cos^{i-1}(\phi) \right]$	$a_1 = 3.7348$ kcal/mol, $a_2 = -4.7925 \times 10^{-1}$ kcal/mol, $a_3 = -7.0492 \times 10^{-1}$ kcal/mol, $a_4 = 1.6780 \times 10^{-1}$ kcal/mol, $a_5 = 5.0437 \times 10^{-1}$ kcal/mol
P1-P1-P1-P2 dihedral		$a_1 = 3.6755$ kcal/mol, $a_2 = -5.6302 \times 10^{-1}$ kcal/mol, $a_3 = -1.1656 \times 10^{-1}$ kcal/mol, $a_4 = 4.1007 \times 10^{-1}$ kcal/mol, $a_5 = -2.3660 \times 10^{-1}$ kcal/mol
P1-P1-P2-P2 dihedral		$a_1 = 3.1646$ kcal/mol, $a_2 = -2.3092 \times 10^{-1}$ kcal/mol, $a_3 = 1.3797$ kcal/mol, $a_4 = 2.1466 \times 10^{-1}$ kcal/mol, $a_5 = -9.9878 \times 10^{-1}$ kcal/mol
P1-P2-P2-P2 dihedral		$a_1 = 3.6582$ kcal/mol, $a_2 = -1.8369 \times 10^{-1}$ kcal/mol, $a_3 = -7.3408 \times 10^{-1}$ kcal/mol, $a_4 = 2.6506 \times 10^{-1}$ kcal/mol, $a_5 = 5.9788 \times 10^{-1}$ kcal/mol
P2-P2-P2-P2 dihedral		$a_1 = 3.5065$ kcal/mol, $a_2 = -3.2871 \times 10^{-1}$ kcal/mol, $a_3 = -6.8060 \times 10^{-2}$ kcal/mol, $a_4 = 1.2472 \times 10^{-1}$ kcal/mol, $a_5 = 1.6408 \times 10^{-1}$ kcal/mol
P2-P1-P1-P2 dihedral		$a_1 = 4.6496$ kcal/mol, $a_2 = 7.0292 \times 10^{-1}$ kcal/mol, $a_3 = -5.8340 \times 10^{-1}$ kcal/mol, $a_4 = 3.3647 \times 10^{-1}$ kcal/mol, $a_5 = -1.1134$ kcal/mol
P1-P2-P1-P1 improper	$U_{\mathrm{improper}}(\chi) = k_\chi \left[\chi - \chi_0 \right]^2$	$k_\chi = 2.1416$ kcal/mol, $\chi_0 = 0°$

Table S2. Nonbonded interactions for P3ATs conjugated polymer. The subscripts 1 and 2 denote the backbone P1 and sidechain P2 beads of P3ATs. In this work, we use $T = 300$ K to derive the corresponding LJ parameters.

**ER functional forms**	**Parameters**
$E_{lj}(r,T) = 4\epsilon(T)\left\{\left[\frac{\sigma(T)}{r}\right]^{12} - \left[\frac{\sigma(T)}{r}\right]^{6}\right\}$	$\epsilon_{11} = \alpha(T) \times 0.199$ kcal/mol, $\sigma_{11} = \beta(T) \times 4.69$ Å<br>$\epsilon_{22} = \alpha(T) \times 0.177$ kcal/mol, $\sigma_{22} = \beta(T) \times 4.69$ Å<br>$\epsilon_{12} = \alpha(T) \times 0.152$ kcal/mol, $\sigma_{12} = \beta(T) \times 4.73$ Å
$\alpha(T) = \frac{\alpha_A - \alpha_G}{1+\exp[-k(T-T_T)]} + \alpha_G$	$\alpha_A = 5.819$, $\alpha_G = 9.214$<br>$k = 3.150 \times 10^{-2}$ K^{-1},$T_T = 310.0$ K
$\beta(T) = \beta_3 T^3 + \beta_2 T^2 + \beta_1 T + \beta_0$	$\beta_3 = 1.451 \times 10^{-9}$ K^{-3}, $\beta_2 = -1.174 \times 10^{-6}$ K^{-2}<br>$\beta_1 = 3.062 \times 10^{-4}$ K^{-1}, $\beta_0 = 1.034$

References

(1) Wang, Y.; Li, Z.; Sun, D.; Jiang, N.; Niu, K.; Giuntoli, A.; Xia, W. Understanding the thermomechanical behavior of graphene-reinforced conjugated polymer nanocomposites via coarse-grained modeling. *Nanoscale* **2023**, *15*, 17124–17137.

(2) Mayo, S. L.; Olafson, B. D.; Goddard, W. A. DREIDING: A Generic Force Field for Molecular Simulations. *J. Phys. Chem.* **1990**, *94*, 8897–8909.

(3) Fortunato, M. E.; Colina, C. M. pysimm: A python package for simulation of molecular systems. *SoftwareX* **2017**, *6*, 7–12.

(4) Alexiadis, O.; Mavrantzas, V. G. All-atom molecular dynamics simulation of temperature effects on the structural, thermodynamic, and packing properties of the pure amorphous and pure crystalline phases of regioregular P3HT. *Macromolecules* **2013**, *46*, 2450–2467.

(5) Gasteiger, J.; Marsili, M. Iterative partial equalization of orbital electronegativity-a rapid access to atomic charges. *Tetrahedron* **1980**, *36*, 3219–3228.

(6) Payne, M. C.; Teter, M. P.; Allan, D. C.; Arias, T. A.; Joannopoulos, J. D. Iterative Minimization Techniques for Abinitio Total-Energy Calculations - Molecular-Dynamics and Conjugate Gradients. *Rev. Mod. Phys.* **1992**, *64*, 1045–1097.

(7) Wang, Y.; Li, Z.; Niu, K.; Xia, W. Energy renormalization for coarse-graining of thermomechanical behaviors of conjugated polymer. *Polymer* **2022**, *256*, 125159.

(8) Chang, T.-M.; Dang, L. X. Liquid-vapor interface of methanol-water mixtures: A molecular dynamics study. *The Journal of Physical Chemistry B* **2005**, *109*, 5759–5765.

(9) Root, S. E.; Savagatrup, S.; Pais, C. J.; Arya, G.; Lipomi, D. J. Predicting the Mechanical Properties of Organic Semiconductors Using Coarse-Grained Molecular Dynamics Simulations. *Macromolecules* **2016**, *49*, 2886–2894.

(10) Tahk, D.; Lee, H. H.; Khang, D.-Y. Elastic moduli of organic electronic materials by the buckling method. *Macromolecules* **2009**, *42*, 7079–7083.

(11) Kleinschmidt, A. T.; Lipomi, D. J. Stretchable Conjugated Polymers: A Case Study in Topic Selection for New Research Groups. *Acc. Chem. Res.* **2018**, *51*, 3134–3143.

(12) Huang, D. M.; Faller, R.; Do, K.; Moulé, A. J. Coarse-grained computer simulations of polymer/fullerene bulk heterojunctions for organic photovoltaic applications. *J. Chem. Theory Comput.* **2010**, *6*, 526–537.

(13) Ding, Z.; Liu, D.; Zhao, K.; Han, Y. Optimizing Morphology to Trade Off Charge Transport and Mechanical Properties of Stretchable Conjugated Polymer Films. *Macromolecules* **2021**, *54*, 3907–3926.
